# Machine-learning screening of luminogens with aggregation-induced emission characteristics for fluorescence imaging

**DOI:** 10.1186/s12951-023-01864-9

**Published:** 2023-03-25

**Authors:** Yibin Zhang, Miaozhuang Fan, Zhourui Xu, Yihang Jiang, Huijun Ding, Zhengzheng Li, Kaixin Shu, Mingyan Zhao, Gang Feng, Ken-Tye Yong, Biqin Dong, Wei Zhu, Gaixia Xu

**Affiliations:** 1 Guangdong Key Laboratory for Biomedical Measurements and Ultrasound Imaging, School of Biomedical Engineering, Health Science Center, Shenzhen University, Shenzhen, Guangdong 518055 China; 2 Key Laboratory of Advanced Textile Materials and Manufacturing Technology and Engineering Research Center for Eco-Dyeing & Finishing of Textiles, Ministry of Education, Zhejiang Provincial Engineering Research Center for Green and Low-carbon Dyeing & Finishing, Zhejiang Sci-Tech University, Hangzhou, 310018 China; 3 School of Biomedical Engineering, The University of Sydney, Sydney, NSW 2006 Australia; 4 Guangdong Provincial Key Laboratory of Durability for Marine Civil Engineering, College of Civil and Transportation Engineering, Shenzhen University, Shenzhen, 518060 China

**Keywords:** Machine learning, Aggregation-induced emission, Artificial intelligence, Fluorescence imaging, Lumiongens

## Abstract

Due to the excellent biocompatible physicochemical performance, luminogens with aggregation-induced emission (AIEgens) characteristics have played a significant role in biomedical fluorescence imaging recently. However, screening AIEgens for special applications takes a lot of time and efforts by using conventional chemical synthesis route. Fortunately, artificial intelligence techniques that could predict the properties of AIEgen molecules would be helpful and valuable for novel AIEgens design and synthesis. In this work, we applied machine learning (ML) techniques to screen AIEgens with expected excitation and emission wavelength for biomedical deep fluorescence imaging. First, a database of various AIEgens collected from the literature was established. Then, by extracting key features using molecular descriptors and training various state-of-the-art ML models, a multi-modal molecular descriptors strategy has been proposed to extract the structure-property relationships of AIEgens and predict molecular absorption and emission wavelength peaks. Compared to the first principles calculations, the proposed strategy provided greater accuracy at a lower computational cost. Finally, three newly predicted AIEgens with desired absorption and emission wavelength peaks were synthesized successfully and applied for cellular fluorescence imaging and deep penetration imaging. All the results were consistent successfully with our expectations, which demonstrated the above ML has a great potential for screening AIEgens with suitable wavelengths, which could boost the design and development of novel organic fluorescent materials.

**Supplementary Information:**

The online version contains supplementary material available at 10.1186/s12951-023-01864-9.

## Introduction

Fluorescent probes, especially organic luminogens, were indispensable agents that were widely used in biological research owing to their lower safety risks and biodegradability[1–3]. However, conventional organic probes still suffer from poor water solubility, severe photobleaching, and low stability. These intrinsic drawbacks significantly hinder the wide applications of organic probes in biomedical research. Fortunately, the luminogens with AIE characteristics (AIEgens) have brought a perfect solution. AIEgens have weak or even no emission at the molecular state but exhibited highly enhanced fluorescence emission in the aggregated state[4]. Such a feature endowed AIEgens with good colloidal stability in water, greater resistance to photobleaching, and highly strengthened functional stability. Therefore, AIEgens have attracted extensive attention among scientists and have been recognized as a better option than conventional organic probes in recent years[5–8].

AIEgens with various wavelengths could be used for various biological applications, such as in vivo fluorescence imaging, orthogonal monitoring, multicolor fluorescence labeling, and fluorescence resonance energy transfer (FRET) analysis[9–12]. In addition, tuning the wavelength of AIEgens could obtain an appropriate penetration depth with minimal interference from tissue absorption, scattering, and autofluorescence to obtain a better signal-to-noise ratio[13]. Researchers showed tremendous interest in designing AIEgens with suitable absorption and emission peaks (λ_abs_, λ_em_). Thus, it is important to have a profound understanding of structure-property relationships between molecular structures and optical properties before chemical synthesis. The challenge is arose from the complex AIE mechanisms based on various dimensions of photophysics, such as restriction of intramolecular rotation or vibration[14], restriction of excited-state deformation[15], suppression of Kasha’s rule[16], and et al. Furthermore, it is noteworthy that the optical properties of molecules are also highly dependent on solvent polarity, which make it even more difficult to design molecules reasonably that match the expected properties[17].

Although some computational methods, such as linear response time-dependent density functional theory (TD-DFT), could be used to predict the λ_abs_ and λ_em_ of molecules, the different hybrid functional and basis sets dramatically impacted on performance[18–20]. For example, in many cases, TD-DFT systematically overestimated the energy of absorption and emission [21, 22]. Although some optimization strategies could address these problems, the computational costs increased significantly[23, 24]. In addition, TD-DFT had significant errors in some skeletons and was unachievable for large-scale screening of molecules given the enormous time complexity. Thus, our aim is to explore a user-friendly approach that only requires information on the molecular structure and solvent to guide us to design and synthesis of molecules.

Among various techniques, machine learning (ML) has grown in popularity and achieved inspiring success in various fields, including drug design, organic synthesis, and materials chemistry due to its time-efficient nature[25–28]. By scanning large datasets and extracting their molecular features, ML models could predict a wide range of properties without understanding the underlying physical or chemical information[29–31]. When there was already some understanding of the physical or chemical mechanism behind it, ML could help provide further insights[32]. This gives researchers the ability to develop molecules with properties that are in accordance with expectations.

**Scheme 1:**
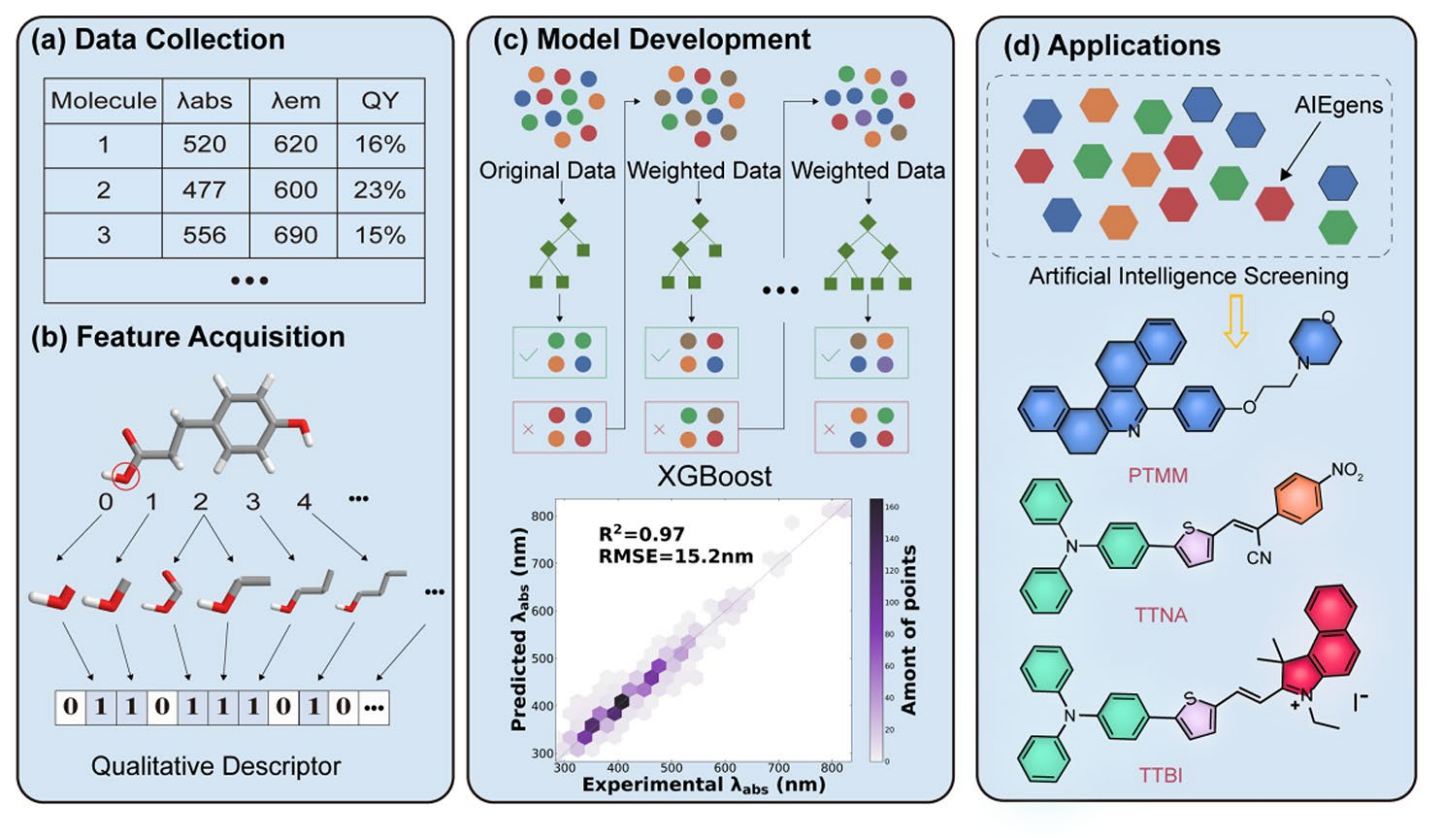
Schematic illustration of (a) data collection, (b) feature acquisition, (c) model development, and (d) applications

In this study, we established a database containing experimental information on 1245 solvated AIEgens, which were collected from the literature published within the last twenty years (24 solvents and 618 AIEgens in various combinations) (Scheme 1a). The molecular structure was first transformed into a vector form that could be recognized by ML (Scheme 1b) and then trained by seven different ML models, including support vector machine (SVM)[33], K-nearest neighbor (KNN)[34], extreme gradient boost (XGBoost)[35], gradient boost regression Tree (GBRT)[36], random forest (RF)[37], multilayer perceptron (MLP)[38] and convolutional neural network (CNN)[39] (Scheme 1c) to predict λ_abs_ and λ_em_. Multi-modal molecular descriptors were further created to improve the accuracy of the ML models for large-scale screening of molecules. Finally, three novel AIEgens with different wavelengths have been predicted and synthesized according to our proposed ML strategy (Scheme 1d). The predicted results were consistent with the experimental results.

## Results and discussion

### Data collection

**Fig. 1:**
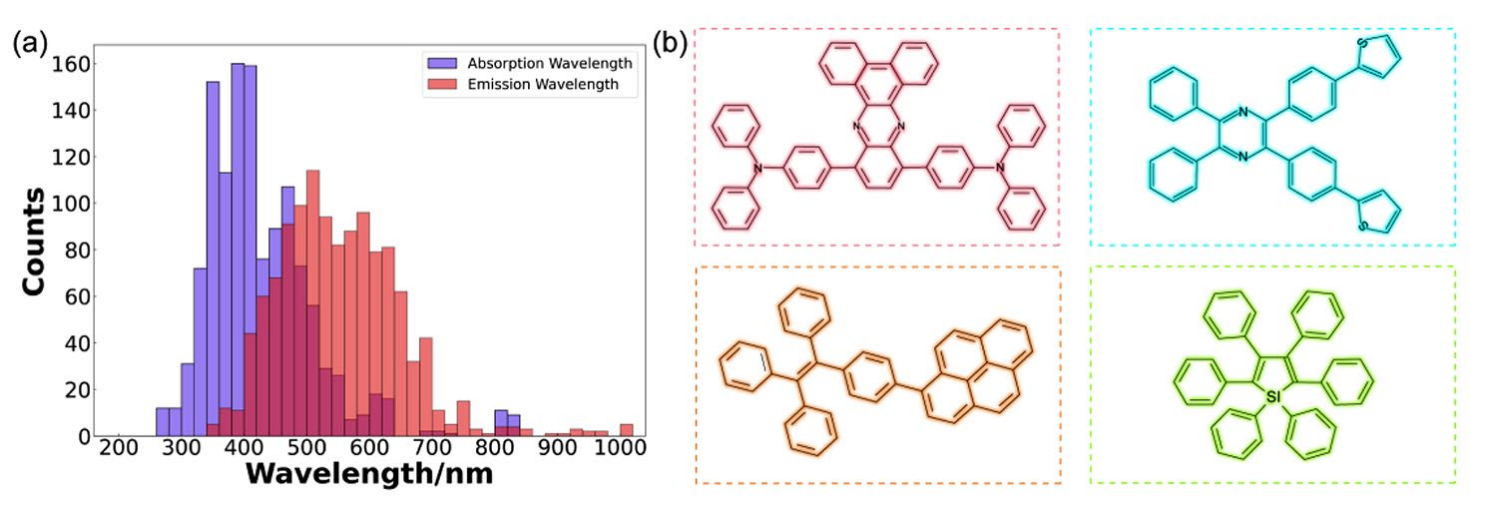
Database information. (a) Data distribution of absorption and emission peaks of solvated AIEgens in our database. (b) Rotor structure of several typical AIEgens in the database

Herein, we created a database containing experimental data of 1245 AIEgens, which were collected from the literature published within the last twenty years (618 distinct AIEgens in 24 solvents). Each data entry contained the molecular structure of AIEgen, solvent, absorption, and emission peaks. If a particular solvated AIEgen has numerous absorption and emission peaks, the absorption peak with the longest wavelength and the emission peak with the highest intensity would be collected. In brief, the absorption and emission peaks of most AIEgens were located in the ranges from visible to the near-infrared region (400 ~ 700 nm) (Fig. 1a). The AIE characteristics of every molecule in the database had been documented in the literature. These AIEgens included rotor structures or their derivatives, such as triphenylamine (TPA), tetraphenylpyrazine (TPP), tetraphenylene (TPE), and hexaphenylsilole (HPS) (Fig. 1b) [4, 40].

### Descriptor Acquisition

**Fig. 2:**
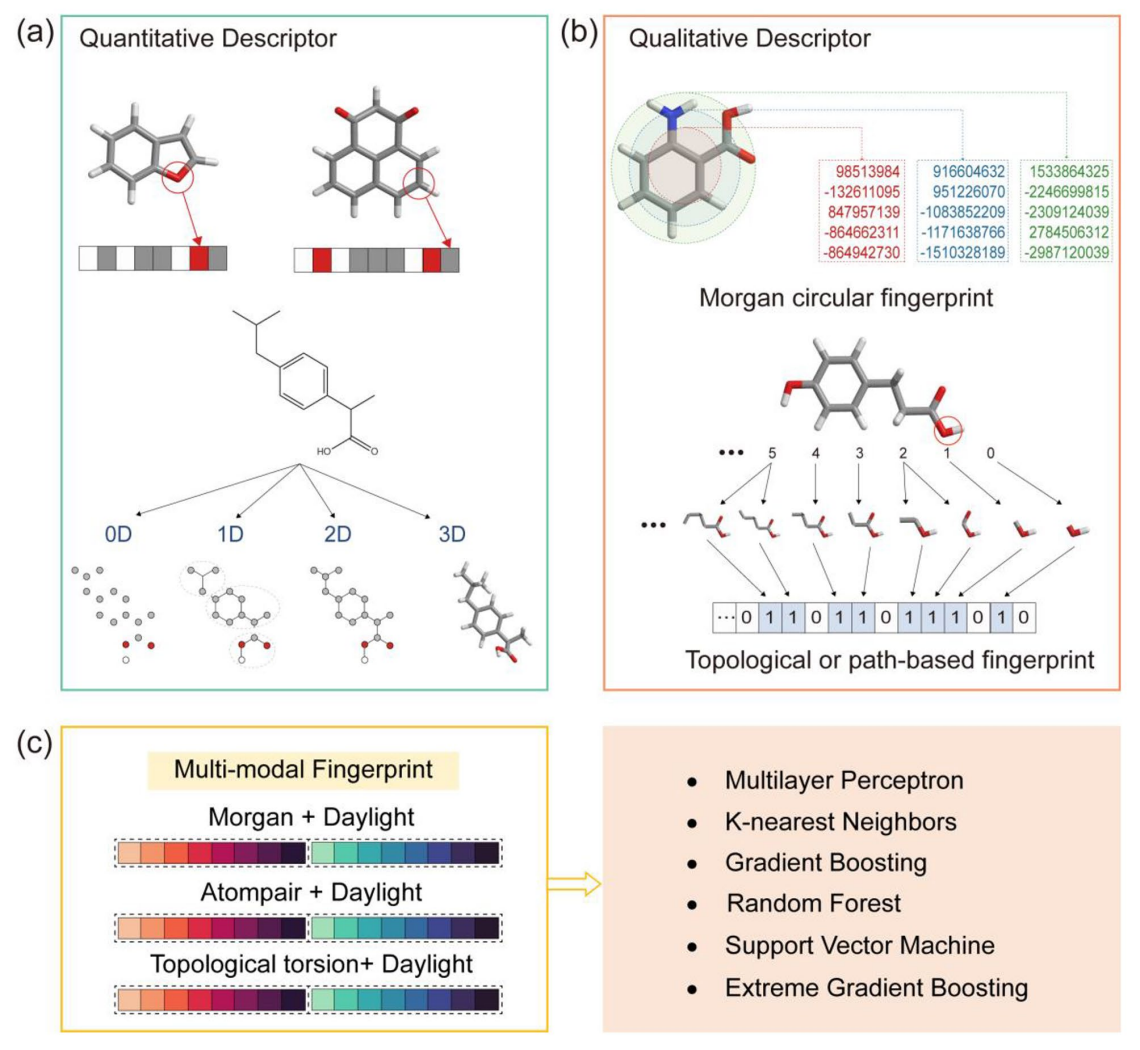
Illustration of the acquisition of molecular descriptors in the study. (a) Quantitative molecular descriptors. (b) Qualitative molecular descriptors. (c) Multi-modal molecular fingerprint for various ML methods

To obtain information that could be recognized and processed by ML, molecules and solvents were converted to molecular descriptors in vector form, that was, descriptor acquisition [41], which was critical to improve the accuracy of the ML models. In this work, we have chosen two forms of molecular descriptors, quantitative (Fig. 2a) and qualitative descriptors (Fig. 2b). The detailed descriptions of molecular descriptors were available in the Molecule descriptors. Quantitative molecular descriptors provided information on a molecule’s physical and chemical properties, such as total molecular weight, lipophilicity, number of electrons, hydrophilicity, hydrophobicity, number of atoms, the fraction of rotatable bonds, and heavy atom molecular weight, etc. Qualitative molecular descriptors were also known as molecular fingerprints. A molecular fingerprint was an abstract representation of a molecule that converted (encoded) it into many bit strings (also known as bit vectors) that were then easily compared between molecules. Each bit on the molecular fingerprint corresponded to the presence or absence of a molecular fragment. We chose Morgan circular fingerprint, Daylight fingerprint, Atom-pair fingerprint, and Topological torsion fingerprint to extract molecular and solvent features. In addition, we created multi-modal molecular descriptors to improve the accuracy of the ML models for large-scale screening of molecules, that was, stitching different types of molecular fingerprints together as a new type of molecular fingerprint (Fig. 2c). Through the use of this strategy, data from various molecular fingerprints were combined to create features that were more complete in their information, increasing the ML model’s accuracy. It has been shown that combining multiple fingerprint features (MFFs, more than 70,000 bits) into a single molecular descriptor had good accuracy[42].

### Model development

**Fig. 3:**
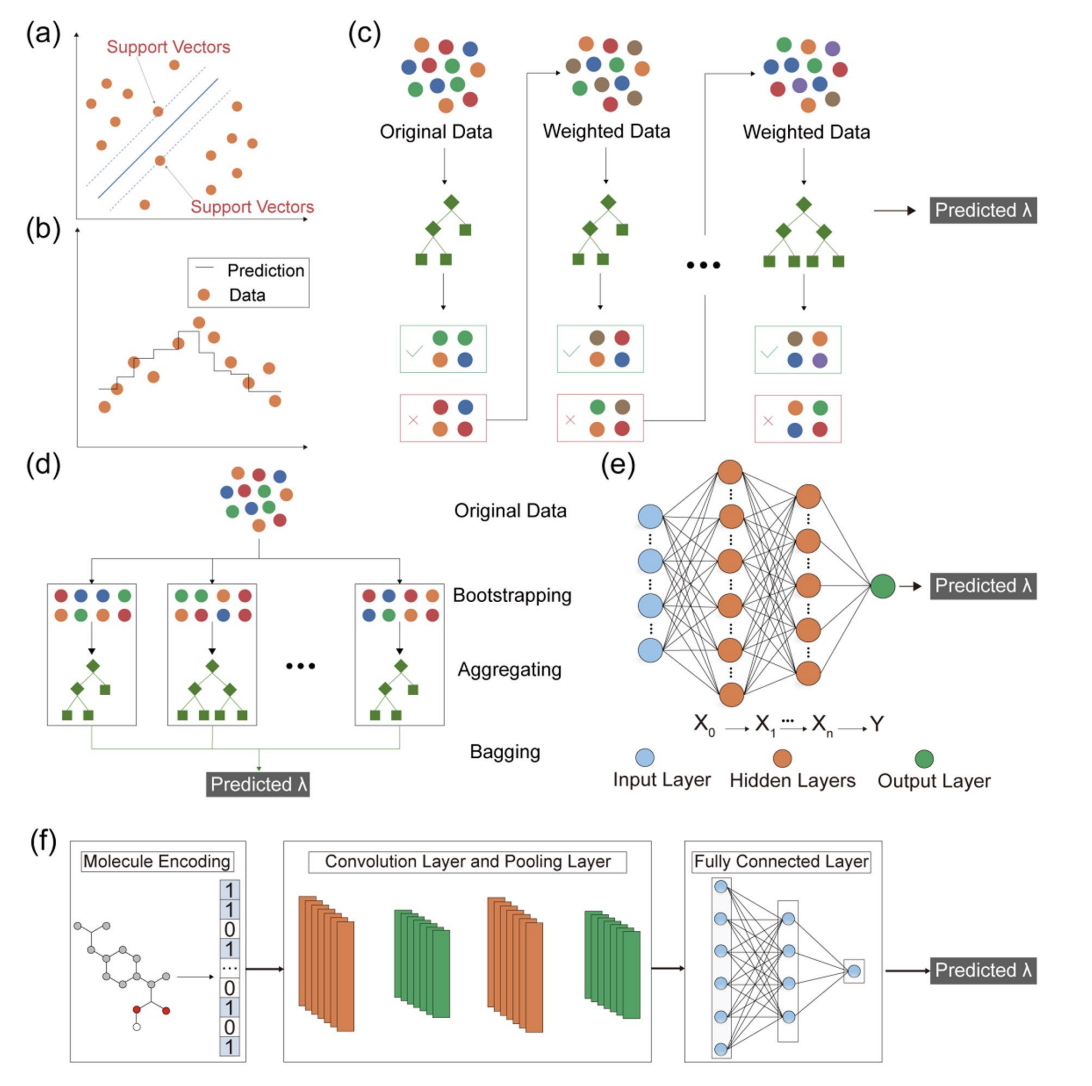
Illustration of the various ML methods used in the study. (a) Support vector machine (SVM). (b) K-nearest neighbor (KNN). (c) Gradient boosting regression tree (GBRT) and extreme gradient boosting (XGBoost). (d) Random Forest (RF). (e) Multilayer perceptron (MLP). (f) Convolutional neural network (CNN).

Model selection was critical for accurate ML prediction, and we have chosen seven state-of-the-art algorithmic models for predicting λ_abs_ and λ_em_. These algorithms were shown in Fig. 3, which included support vector machine (SVM), K-nearest neighbor (KNN), extreme gradient boost (XGBoost), gradient boost regression Tree (GBRT), random forest (RF), multilayer perceptron (MLP) and convolutional neural network (CNN). To compare and assess the effectiveness of the algorithms, we used the mean absolute error (MAE) as an evaluation metric. Furthermore, we adopted a 10-fold cross-validation strategy to evaluate different methods under different molecular descriptors (Figure S22).

**Fig. 4:**
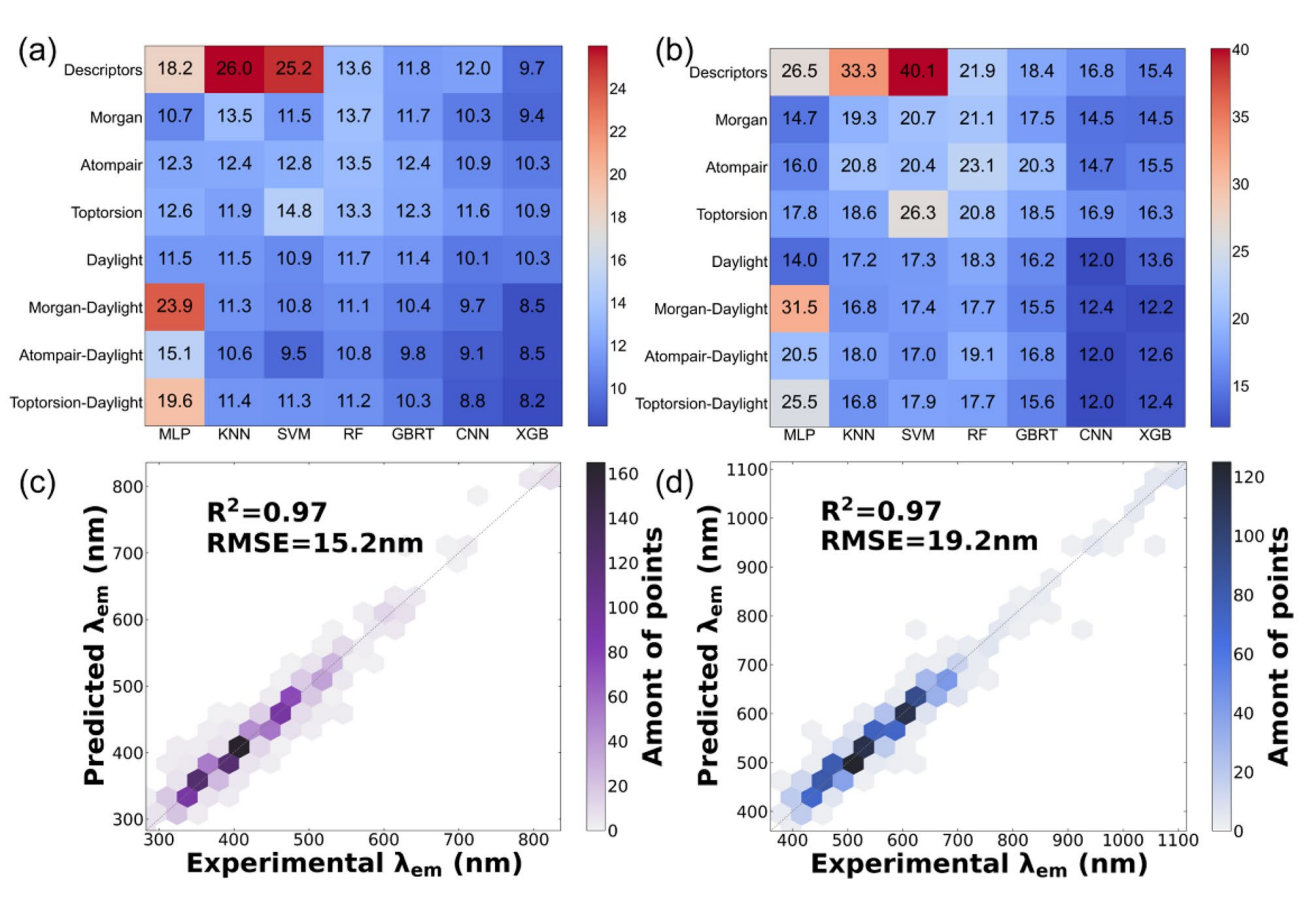
Testing results of absorption and emission wavelengths of ML models with various molecular descriptors using 10-fold cross-validation. (a) MAE of predicted absorption peak. (b) MAE of predicted emission peak. (c) The XGB model predicts λ_abs_ based on toptorsion-daylight fingerprint. (d) The CNN model predicts λ_em_ based on atom-pair-daylight fingerprint

To evaluate the performance of different combinations of these molecular descriptors and ML algorithm models, we first compared the performance of ML on the test set. Both absorption (Fig. 4a) and emission (Fig. 4b) predictions were evaluated. The outcomes showed that multi-modal molecular descriptors consistently performed better than single molecular fingerprints, demonstrating the superiority and robustness of our proposed multi-modal molecular descriptors strategy. Multilayer perceptron (MLP) is one type of neural network that has recently attracted tremendous attention among researchers. However, since neural network need more information to function better, the MLP model hasn’t done the best on our dataset. Additionally, the MLP model performed poorly on multi-modal molecular descriptors, likely the result of overfitting due to the MLP model’s sensitivity to high-dimensional data. RF was a general ensemble learning algorithm that produced the final decision by combining the results of individual trees constructed on a randomly chosen subset of data. This strategy for combining various sub-results into a final result did not produce significant errors and was thus stable on our dataset. However, RF did not perform as well on regression problems as it did on classification problems because it could not produce predictions beyond the scope of the data in the training set, resulting in poorer results than other models. In terms of qualitative molecular descriptors, the SVM model and KNN model performed better than the RF model but worse than quantitative molecular descriptors. This might be because qualitative molecular descriptors were better suited for our dataset, and the same trend could be seen for the MLP model. The CNN and XGB models outperformed the others for single and multi-modal molecular descriptors. CNN was a popular image processing model for extracting multi-dimensional feature information from images. We adopted molecular fingerprints in vector form to the CNN model in this work. We obtained superior results because the topological information generated by molecular fingerprints could be extracted maximally by the one-dimensional convolutional kernel. The XGB model was based on the cumulative, iterative GBRT model and enhanced it. Both XGB and GBRT were ensemble methods based on regression trees. Thus, they had excellent performance because they were less likely to be overfitting or underfitting. For molecular fingerprints, Daylight fingerprints generally outperformed other fingerprints on various models because Daylight fingerprints express molecular information in a topological manner that was more suitable for the structure of AIEgens.

The XGB model performed better at predicting λ_abs_, while the CNN model excelled at predicting λ_em_. To determine the most applicable multi-modal molecular descriptors for these two models, two additional evaluation metrics, coefficient of determination (R^2^) and root mean square error (RMSE), were added to determine further the level of fit and magnitude of error between ML predictions and experimental results. As shown in Fig.S18, we assessed how well the two models performed for each of the three multi-modal molecular descriptors. From the results, the scatter plot and R^2^ showed that our model performed equally well on various multi-modal molecular descriptors, with an R^2^ of 97%. For the RMSE values, the toptorsion-daylight fingerprint was most suitable for the XGB model for predicting absorption peaks (Fig. 4c), and the atompair-daylight fingerprint was ideal for the CNN model predicting emission peaks (Fig. 4d). Furthermore, ML performed better in absorption peak prediction, which was understandable given the structure-property relationship and the solvation effect of AIEgens. However, given the significance of fluorescence emission in biological applications, we considered that the emission prediction was more important.

**Fig. 5:**
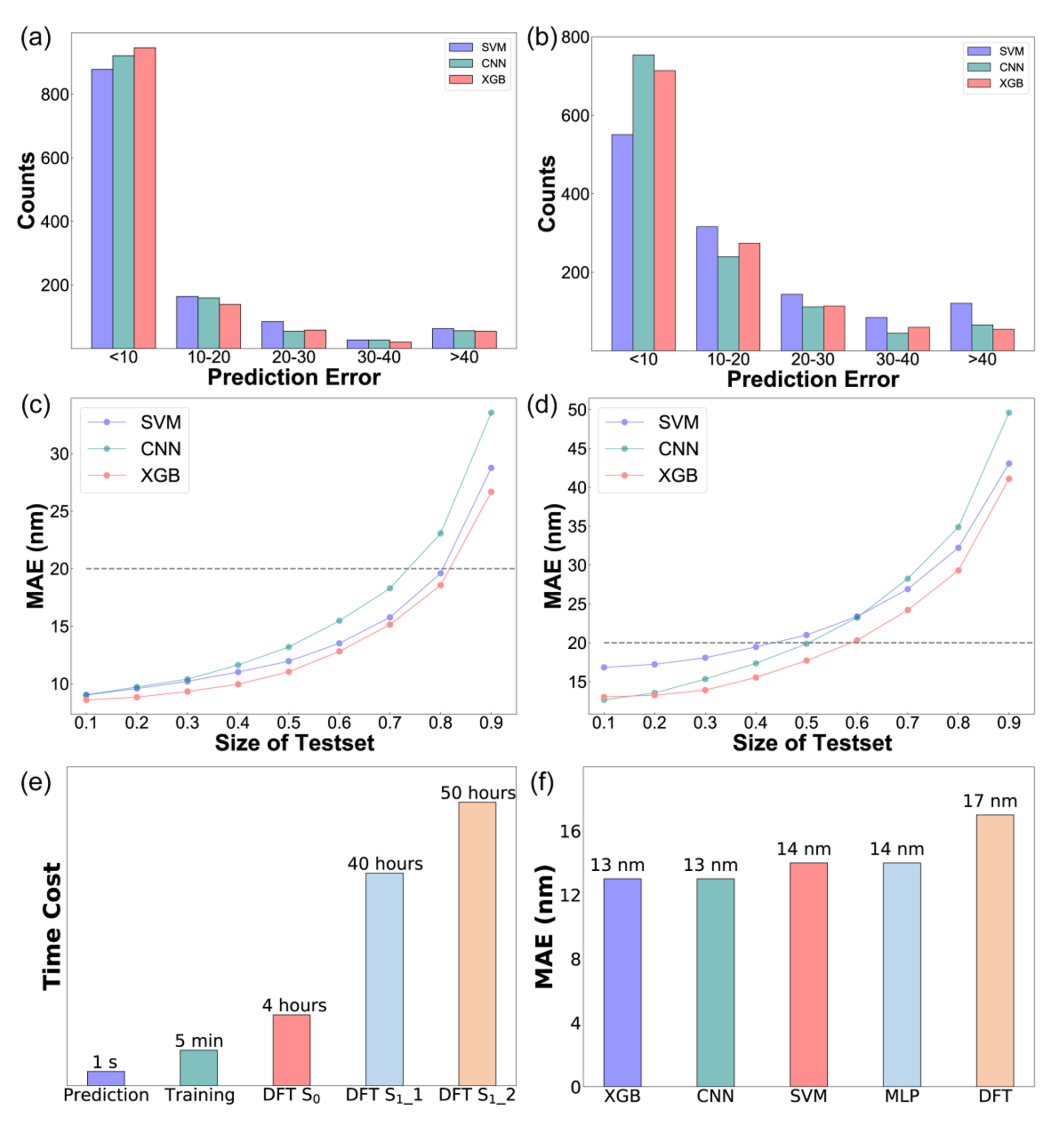
ML prediction error distribution, model scalability, and comparison of ML and TD-DFT. (a) Absorption peak error distribution. (b) Emission peak error distribution. (c) Absorption model scalability. (d) Emission model scalability. (e) Time cost for one molecule. (f) MAE of predicted absorption peak

With the above results, we have initially constructed and screened ML models with excellent performance. We have also analyzed our models’ error distribution and scalability in screening AIEgens with the desired optical properties (Fig. S19 and S20). As shown in Fig. 5a and b, the predictions achieved by 10-fold cross-validation had the majority of prediction errors within 10 nm, with the number of errors greater than 20 nm accounting for around 10% of the total, demonstrating that our model had excellent performance. In general, the better performance of ML models was obtained, the more data were included. On our dataset, we investigated the model’s ability to scale, that was, how the model would perform as the size of the test set and training set were gradually increased and decreased, respectively. As the test set was reduced and as the training set was expanded, the MAE of these models gradually declined, and their performance gradually improved (Fig. 5c and d).

To validate the utility of ML, we compared it to TD-DFT, a method that could calculate molecules’ absorption and emission peaks (Fig. S21). We gathered 36 AIEgens from the literature that contained TD-DFT calculations. All of the AIEgens we collected used a theory level of B3LYP/6-31G(d) to avoid the effects of different basis sets and functionals on the results. As illustrated in Fig. 5e, it consumed longer time by using the TD-DFT method than using ML. According to statistics, it took approximately 4 h to optimize the molecular ground state structure, about 40 h to optimize the molecular excited state structure, and about 50 h to calculate the molecular single point energy based on the optimized molecular excited state structure[43]. In contrast, it took no more than 5 min to train the ML models on our server, and the trained ML models predicted the molecule in less than 1 s. As a result, the time-cost issue with TD-DFT was resolved by using ML as an alternative, significantly increasing the efficiency of large-scale screening of AIEgens with expected absorption and emission wavelength. As shown in Fig. 5f, not only did ML outperform TD-DFT in terms of time cost, but the accuracy of the ML model was also higher than the calculated results of TD-DFT. The reason for the poorer outcomes of TD-DFT was that TD-DFT tended to overestimate the absorption and emission energies of molecules, resulting in relatively large errors[21, 22]. In addition, TD-DFT had significant errors in some skeletons and the results were much worse than those generated by ML models[44]. In contrast to quantum chemical calculations, trained ML models allowed researchers to quickly and accurately obtain results without the need for extensive knowledge of physics, chemistry, or quantum computing. Therefore, ML may overtake first-principles calculations as chemists’ preferred method in the future.

In this section, we evaluated various ML models and molecular descriptors for predicting the absorption and emission peaks of AIEgens in various solvents. Two combinations with optimal performance were developed to meet the needs of large-scale screening. The XGB model with toptorsion-daylight fingerprint performed best in predicting absorption peaks, and the CNN model with atom-pair-daylight fingerprint performed best in predicting emission peaks. We considered several evaluation metrics, error distributions, and model scalability in our evaluation process. Furthermore, it has been demonstrated that our ML models could improve performance by using multi-modal molecular descriptors and expanding the database. These results showed the viability of our ML model in practical applications.

### Synthesis and application according to the predictions

**Fig. 6:**
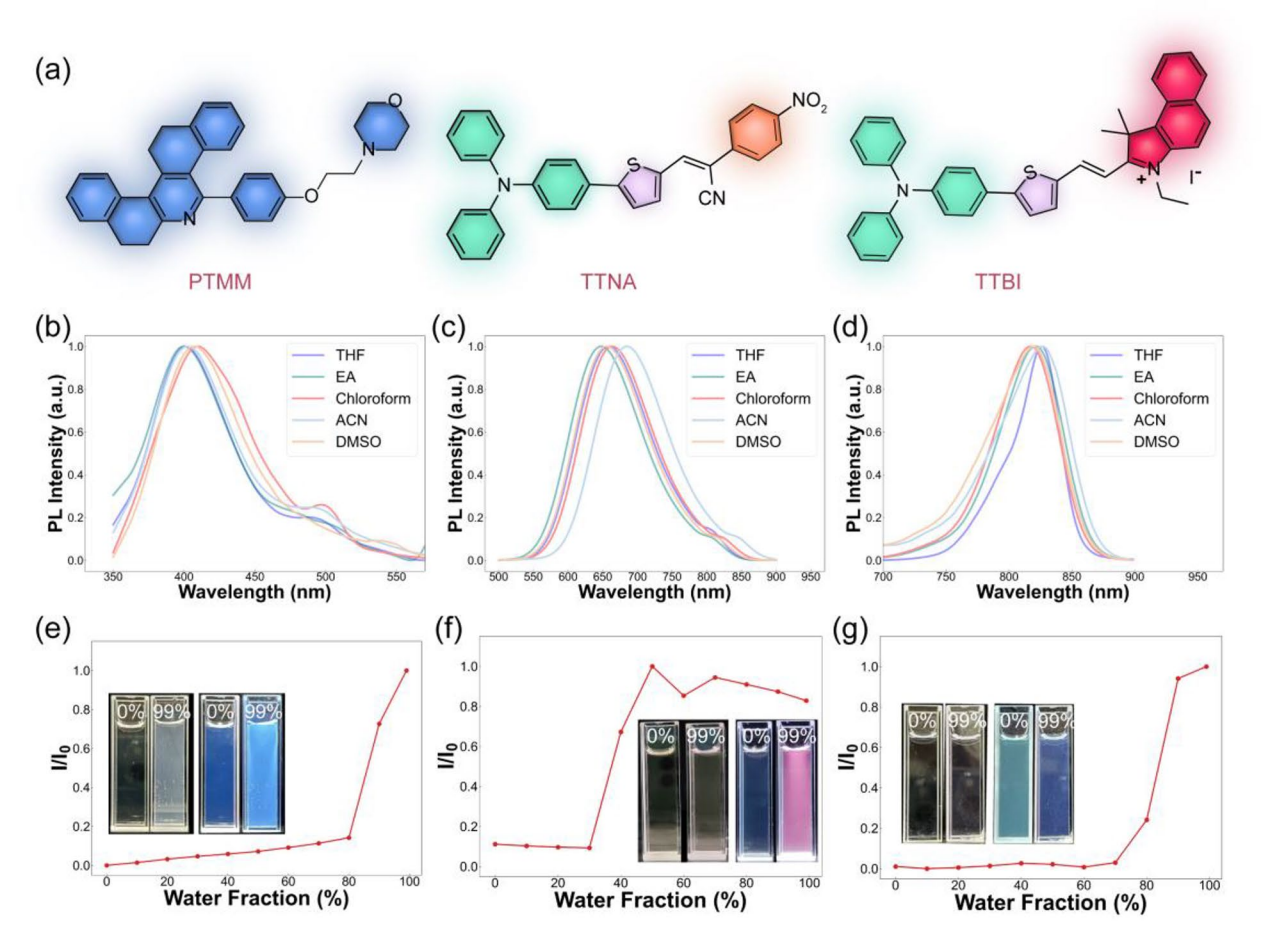
Optical properties of PTMM, TTNA, and TTBI. (a) Chemical structure of PTMM, TTNA, and TTBI. (b) Normalized Photoluminescence (PL) spectra of (b) PTMM, (c) TTNA, and (d) TTBI in various solvents. PL intensities of (e) PTMM, (f) TTNA, and (g) TTBI in ACN/water mixtures with different volume fractions of water. Inset: Digital images of corresponding AIEgens exposed to white and UV radiation (365 nm) with different water fractions

To validate the ability of the ML model to predict the structure of novel molecules, we synthesized a series of potential AIEgens at different wavelengths based on the results of ML screening and their synthesizability (Scheme S1). The molecular structures of these AIEgens were depicted in Fig. 6a, PTMM was simply synthesized by the one-pot reaction of 2-tetralone and benzaldehyde derivative, and the non-coplanar backbone between pyridine core and benzene ring endow the molecules with active intramolecular torsion. Then TTNA and TTBI were facilely synthesized through a few-step-reactions, which are both comprised of triphenylamine moiety (working as donor), thiophene fragment (D and π-bridge), double bond (π-bridge) and the nitrobenzene/ quaternary ammonium salt unit (A). Additionally, to validate the ability of our model to predict the absorption and emission peaks of AIEgens in different solvents, we measured the absorption (Fig. S12) and emission (Fig. 6b-d) spectra of these AIEgens in five different solvent systems, including tetrahydrofuran (THF), ethyl acetate (EA), chloroform (Chloroform), acetonitrile (ACN), and dimethyl sulfoxide (DMSO). It can be seen that the peaks of the PL spectra of the three AIEgens show some differences with increasing solvent polarity. Suggesting a certain solvent effect and strong twisted intramolecular charge transfer (TICT) effect[45]. The AIE characteristics of three AIEgens were further examined using PL spectra in different ACN/water solvent systems (Fig. 6e-g), when the water fraction continuously increased, the fluorescence intensity of the AIEgens enhanced largely, which was attributed to the mechanism of restriction of intramolecular motions along with aggregation[46]. In such a binary solvent system, there is a competition between AIE and TICT on the PL.

For both the absorption peaks and emission peaks of the three molecules, as shown in Table S1, the experimental results were in good agreement with the ML-predicted outcome at five different solvents, which demonstrated that our ML models could accurately predict the absorption and emission peaks of AIEgens in different solvents at various wavelengths.

**Fig. 7:**
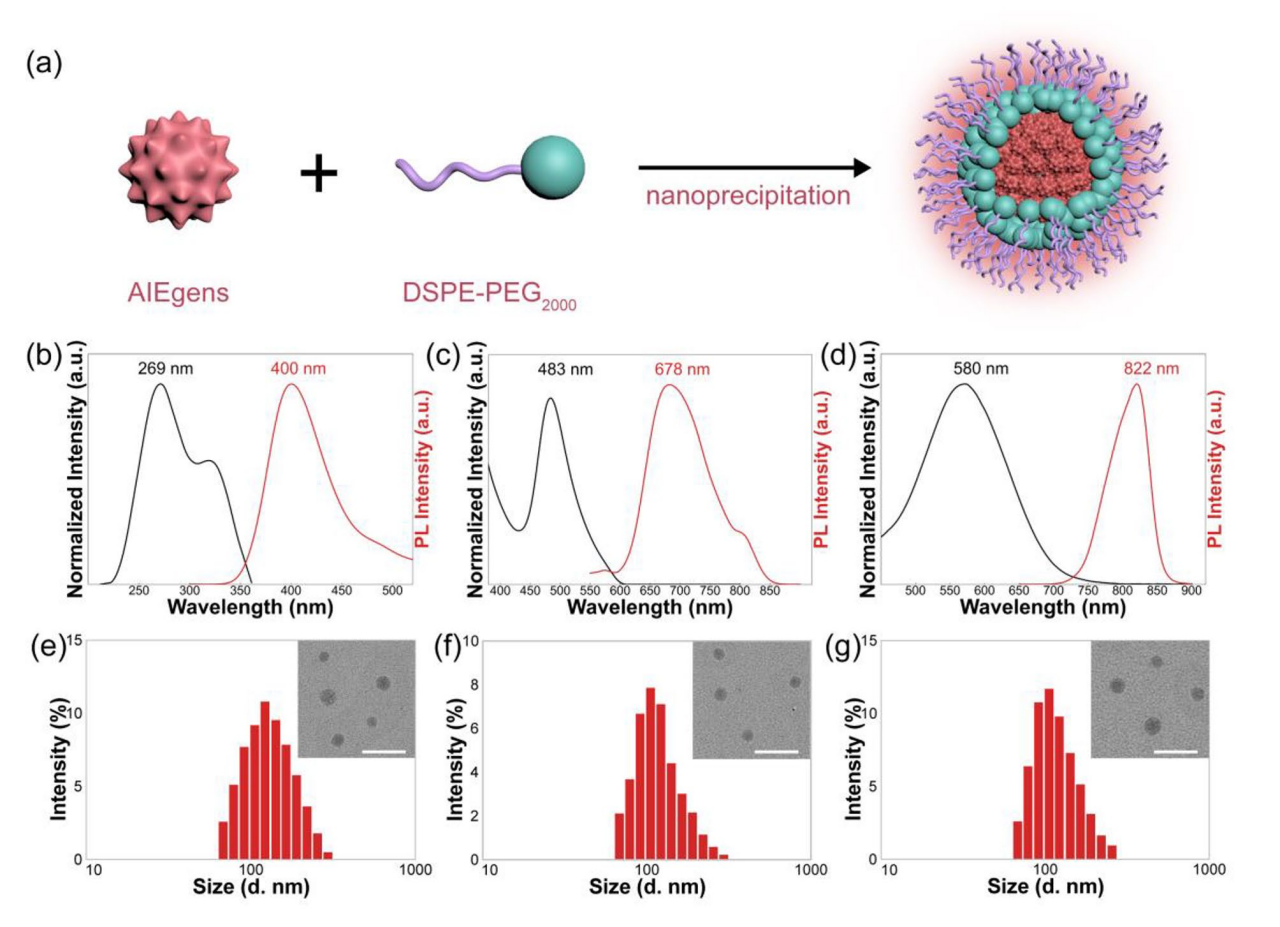
Optical properties of Nanoparticles (NPs). (a) Schematic illustration of NPs fabrication by nanoprecipitation. Normalized absorption and fluorescence spectra of (b) PTMM NPs, (c) TTNA NPs, and (d) TTBI NPs in aqueous solutions. DLS profile of (e) PTMM NPs, (f) TTNA NPs, and (g) TTBI NPs. Inset: TEM images of corresponding NPs (Scale bar, 200 nm)

To confirm that the AIEgens predicted by ML model could be used for biomedical applications, AIEgens were firstly encapsulated into nanoparticles (NPs) by nanoprecipitation using DSPE-PEG_2000_ as an encapsulation matrix (Fig. 7a). The morphology and photophysical properties of PTMM NPs, TTNA NPs, and TTBI NPs were investigated. As shown in Fig. 7b-d, the absorption peaks for PTMM NPs, TTNA NPs, and TTBI NPs in water were observed at 269, 483, and 589 nm, respectively. The emission peaks for these NPs in water were observed at 400, 678, and 822 nm, respectively. The hydrodynamic sizes of the PTMM NPs, TTNA NPs, and TTBI NPs were measured by dynamic light scattering (DLS). As illustrated in Fig. 7e-g, the average diameter of PTMM NPs was ~ 120 nm, TTNA NPs was ~ 90 nm, and TTBI NPs was ~ 100 nm. The transmission electron microscope (TEM) images showed that the average sizes of the formed PTMM NPs, TTNA NPs, and TTBI NPs were close to 80, 60, and 70 nm, respectively.

**Fig. 8:**
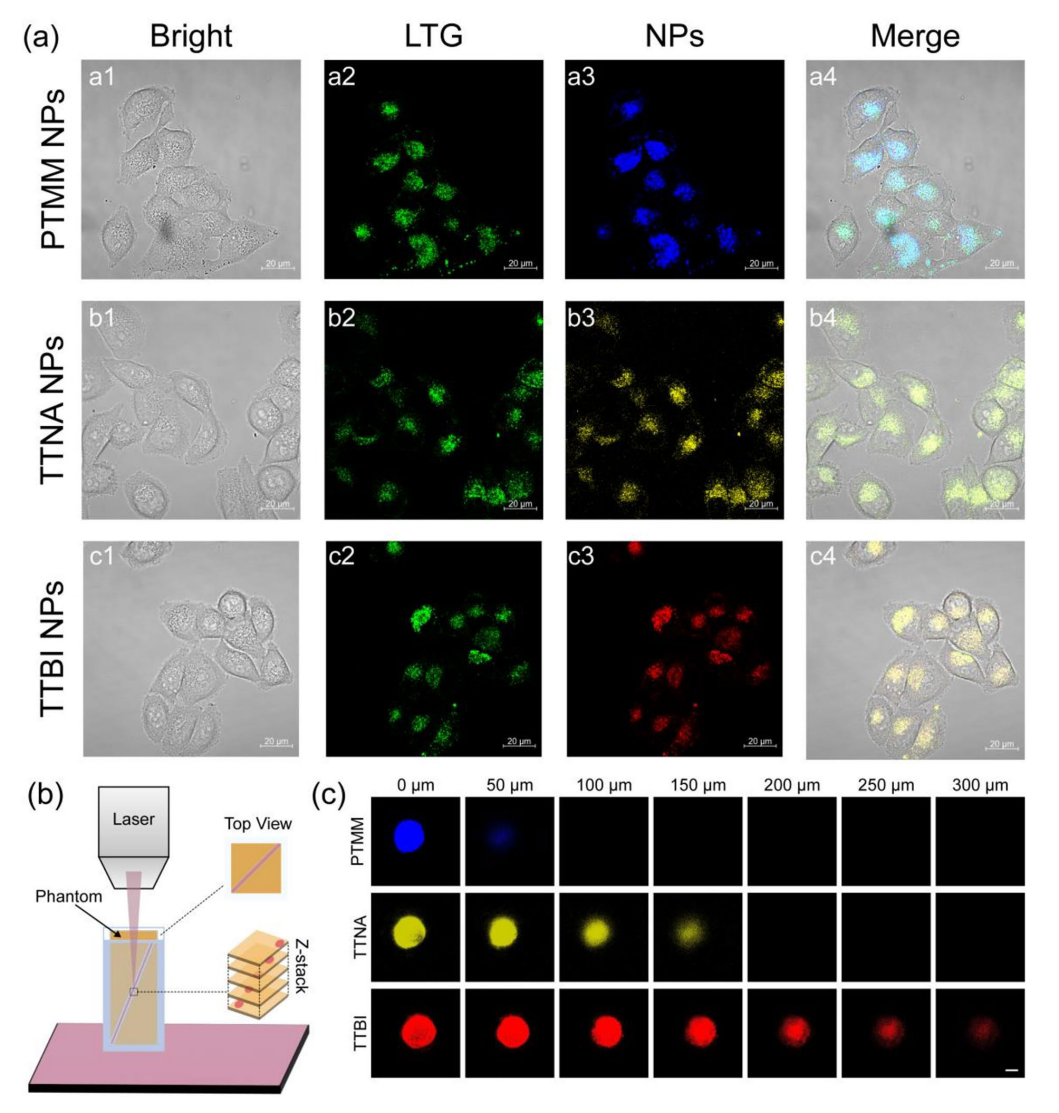
Cell imaging, co-localization imaging and tissue penetration depth evaluated by a phantom model. Confocal images of HeLa cells after co-culture with NPs for 4 h, including bright field channel, Lysosome-Tracker Green (LTG) channel, NPs channel, and merged image. (a1-a4) PTMM NPs; (b1-b4) TTNA NPs; (c1-c4) TTBI NPs. Scale bar: 20 μm. (b) Schematic illustration of evaluation of tissue penetration by a phantom model. (c) Z-stack images of phantom with intervals of 50 μm. Scale bar: 200 μm

In order to investigate the biocompatibility and stability of the AIEgens NPs for biomedicine, a cell labeling experiment was performed. HeLa cells were co-cultured with PTMM NPs, TTNA NPs, and TTBI NPs and a confocal laser scanning microscope (CLSM) was applied to take the fluorescence images. Considering the general uptake pathway of NPs, lysosome-tracker green (LTG) probe was applied to localize the AIEgens NPs[47, 48]. As shown in Fig. 8a3-c3, an intense blue and red fluorescent signal was observed, indicating a good signal-to-noise ratio within the cells. As shown in Fig. 8a4-c4, the merged images of two fluorescence channels overlapped perfectly, demonstrating that PTMM NPs, TTNA NPs, and TTBI NPs entered the cellular lysosomes after 4 h co-culture. The results showed that our ML model performed superbly in predicting new structures and that the AIEgens identified through ML screening could be successfully used in biological applications.

Furtherly, to evaluate the deep tissue imaging capability of AIEgens NPs with various emission peaks, phantom models were prepared to simulate skin tissue (Fig. 8b). A quartz capillary loaded with different NPs was inserted into the phantom for fluorescence imaging, and the results are shown in Fig. 8c. The results suggested that stronger signals could be seen at deeper penetration depths using AIEgens with long absorption and emission wavelengths as we expected.

## Conclusions

In this study, we created a dataset of solvated AIEgens gathered from the literature. Five molecular descriptors, including the morgan circular fingerprint, daylight fingerprint, atom-pair fingerprint, topological torsion fingerprint, and quantitative descriptors were chosen and used to decipher the molecular structure and solvent properties. By combining the proposed multi-modal molecular descriptor approaches with various ML models, we have obtained good and reliable predictive results. This strategy took into account the properties of different molecular descriptors, not only learning the structural details of existing molecules but also accurately predicting the properties of unknown molecules. Three novel AIEgens were then predicted and synthesized according to the large-scale ML screening. Remarkably, good consistency between the predictive and experimental results has been obtained. The screened AIEgens were further applied in the cellular fluorescence imaging and the deep penetration imaging. All the results were consistent with our expectations. In this new paradigm, we provided a novel ML method for new AIEgens design with desired optical properties and dramatically less time cost, thereby boosting the development of high-performance organic fluorescent materials.

## Experimental section

### Materials and instruments

4-Hydroxybenzaldehyde, 4-(2-chloroethyl)morpholine, 2-tetralone, ammonium acetate, 4-bromo-N,N-diphenylaniline, (5-formylthiophen-2-yl)boronic acid, PdCl_2_(dppf), 1,1,2-trimethyl-1 H-benzo[e]indole, 2-(4-nitrophenyl)acetonitrile, iodoethane and solvents were all purchased from Sigma Aldrich and used as received without further purification. Chloroform and ethanol was obtained from Macklin reagent. 2-Distearoyl-sn-glycero-3-phosphoethanolamine-N-[methoxy(polyethylene glycol)-2000 (DSPE-mPEG2000) was purchased from Xi’an ruixi Biological Technology Co., Ltd. PBS (pH 7.4) was purchased from Beyotime Biotechnology and Lyso-tracker Green were purchased from Sigma Aldrich. DMEM medium, fetal bovine serum (FBS), penicillin and streptomycin were purchased from Gibco. 3-Ethyl-1,1,2-trimethyl-1 H-benzo[e]indol-3-ium iodide was synthesized according to the literature method[49].

^1^ H and ^13^ C NMR spectra were recorded with a Bruker ARX 400 NMR spectrometer using CDCl_3_ or DMSO-*d*_*6*_ as solvent. Liquid Chromatography-Mass Spectrometry (LC-MS) was recorded on a Thermo Scientific LCQ Fleet. High-resolution mass spectra (HRMS) were recorded on XEVO G2-XS QTOF Mass Spectrometer System operating in a Matrix-Assisted Laser Desorption/Ionization Time of Flight (MALDI-TOF) mode. UV–vis absorption spectra were measured on a PerkinElmer Lambda 950 spectrophotometer. Photoluminescence (PL) spectra were recorded on Edinburgh FS5 fluorescence spectrophotometer. The particle size and zeta potential were measured using a Malvern Zetasizer Nano-ZS90. The particle size and morphology were observed on a HITACHI-HT7700 transmission electron microscope (TEM). Confocal laser scanning microscopy (CLSM) images were collected on a ZEISS-LSM880 CLSM. The chemical structures of the final products have been confirmed by NMR spectra and mass spectra (Figures S1–S11, Supporting Information).

### Synthesis of PM[50]

To a mixture of 3-hydroxybenzaldehyde (1.0 g, 8.20 mmol) in acetonitrile were added 4-(2-chloroethyl)morpholine (1.2 g, 8.20 mmol) and anhydrous potassium carbonate (1.68 g, 12.3 mmol), and the mixture was refluxed for 10 h. The mixture was filtered and dried to give the crude product. The crude product was finally purified by column chromatography (DCM/MeOH = 50:1) to yield the desired compound as brown oil (1.39 g, yield 72%). ^1^ H NMR (500 MHz, CDCl_3_ ) δ 9.88 (s, 1 H), 7.90–7.77 (m, 2 H), 7.09–6.94 (m, 2 H), 4.20 (t, *J* = 5.7 Hz, 2 H), 3.80–3.67 (m, 4 H), 2.84 (t, *J* = 5.6 Hz, 2 H), 2.67–2.49 (m, 4 H).

### Synthesis of PTMM[51]

In a 50 mL round-bottomed flask, added ammonium acetate (0.77 g, 10 mmol), **PM** (0.24 g, 1.0 mmol), 2-tetralone (0.30 g, 2.0 mmol), 10 mL of glacial acetic acid and stirred for 24 h at room temperature. After the completion of the reaction as monitored by TLC, the resulting product was poured into water. The solid was obtained, filtered, and purified by column chromatography (PE/EA = 10:1) as eluent to give the light green solid (0.42 g, yield: 85%). ^1^ H NMR (400 MHz, CDCl_3_) δ 7.58–7.50 (m, 1 H), 7.46–7.36 (m, 3 H), 7.35–7.31 (m, 2 H), 7.37–7.29 (m, 1 H), 7.28 (s, 1 H), 7.03–6.93 (m, 2 H), 6.94–6.88 (m, 2 H), 4.20 (t, *J* = 5.8 Hz, 2 H), 3.81 (t, *J* = 4.4 Hz, 4 H), 3.21–3.09 (m, 4 H), 3.03–2.95 (m, 2 H), 2.88 (s, 2 H), 2.80 (t, *J* = 6.5 Hz, 2 H), 2.66 (s, 4 H). ^13^ C NMR (101 MHz, CDCl_3_) δ 158.48, 158.13, 153.52, 145.75, 138.72, 133.28, 131.18, 129.54, 128.70, 127.89, 127.61, 127.22, 126.91, 126.07, 125.72, 114.56, 66.87, 65.79, 57.69, 54.15, 33.27, 29.62, 29.55, 29.32. LC-MS : m/z, cal 488.246, found: 489.521 [M + H]^+^; Retention time = 0.881 min.

### Synthesis of TTA[52]

A solution of 4-bromo-N,N-diphenylaniline (1.0 g, 3.0 mmol) and (5-formylthiophen-2-yl)boronic acid (0.63 g, 4.0 mmol) was refluxed under nitrogen in the mixed toluene/MeOH (20 mL: 20 mL) in the presences of PdCl_2_(dppf) (0.23 g, 0.31 mmol) and K_2_CO_3_ (2.13 g, 15.4 mmol) for 24 h. The combined organic phase was filtered and dried to obtain the crude product, which was further purified by silica-gel chromatography (PE/DCM = 2:1) to obain the yellow solid (0.74 g, yield: 69.2%). ^1^ H NMR (400 MHz, CDCl_3_) δ 9.88 (s, 1 H), 7.73 (d, *J* = 3.9 Hz, 1 H), 7.57–7.52 (m, 2 H), 7.35–7.29 (m, 5 H), 7.19–7.14 (m, 4 H), 7.14–7.06 (m, 4 H).

### Synthesis of TTNA[53]

A solution of 5-(4-(diphenylamino)phenyl)thiophene-2-carbaldehyde (0.177 g, 0.5 mmol) and 2-(4-nitrophenyl)acetonitrile (0.810 g, 0.5mmol) were added to ethanol (20 mL) with a drop of piperidine and refluxed for 5 h. It was then cooled down to room temperature and produced a black product, which was then filtered, washed three times with cold ethanol, and dried in a vacuum (0.167 g, 67%). ^1^ H NMR (500 MHz, CDCl_3_) δ 8.32–8.26 (m, 2 H), 7.85–7.75 (m, 3 H), 7.65 (d, *J* = 4.0 Hz, 1 H), 7.57–7.52 (m, 2 H), 7.34–7.26 (m, 5 H), 7.17–7.11 (m, 4 H), 7.11–7.03 (m, 4 H). ^13^ C NMR (126 MHz, CDCl_3_) δ 151.92, 149.07, 147.42, 147.12, 140.66, 137.34, 136.64, 135.48, 129.62, 127.28, 126.28, 126.10, 125.27, 124.55, 123.99, 122.99, 122.61, 117.84, 103.97. MALDI-TOF (ESI): m/z calcd for C_31_H_21_N_3_O_2_S [M]^+^,499.1354; found, 499.1354.

### Synthesis of TTBI

The synthetic procedure for the preparation of **TTBI**[52]. A solution of 5-(4-(diphenylamino)phenyl)thiophene-2-carbaldehyde (0.1 g, 0.3 mmol) and 3-ethyl-1,1,2-trimethyl-1 H-benzo[e]indol-3-ium iodide (0.13 g, 0.36 mmol) was refluxed in dry ethanol catalyzed by a few drops of piperidine for 10 h under nitrogen. After cooling to room temperature, the solvent was evaporated under reduced pressure. The residue was purified by silica-gel chromatography (DCM/MeOH = 20:1) to give the purple-black solid (0.19 g, yield: 88.4%). ^1^ H NMR (400 MHz, CDCl_3_) δ 8.70 (d, *J* = 15.5 Hz, 1 H), 8.51 (d, *J* = 4.1 Hz, 1 H), 8.25 (d, *J* = 8.4 Hz, 1 H), 8.15–8.05 (m, 3 H), 7.85–7.62 (m, 3 H), 7.59 (d, *J* = 8.4 Hz, 2 H), 7.45 (s, 1 H), 7.37–7.32 (m, 4 H), 7.17 (t, *J* = 7.6 Hz, 6 H), 7.07 (d, *J* = 7.2 Hz, 2 H), 4.90 (m, *J* = 7.5 Hz, 2 H), 2.17 (s, 6 H), 1.48 (t, *J* = 7.3 Hz, 3 H). ^13^ C NMR (101 MHz, CDCl_3_) δ 180.19, 157.17, 149.94, 145.76, 142.12, 137.84, 133.42, 131.70, 130.29, 129.64, 128.57, 127.69, 125.60, 124.43, 122.78, 121.67, 111.74, 107.20, 46.27, 27.18, 22.69. LC-MS : m/z, cal 575.252, found: 575.552 [M]^+^; Retention time = 0.979 min.

### Synthesis of NPs

Fabrication of NPs was carried out by injecting THF solution (0.5ml) of AIEgens (1 mg) and DSPE-PEG_2000_ (5 mg) into 5ml of ultrapure water and stirring vigorously for 2 min. The prepared NPs were purified for a day using ultrapure water dialysis (molecular weight cutoff of 100 kDa). After that, NPs were ultrafiltered for 20 min at 4400 rpm through ultrafiltration tubes with a molecular weight cutoff of 100 kDa. After ultrafiltration, the NPs were dispersed in 1°x PBS buffer (pH 7.4) and kept out of the light at 4 °C.

### Cell culture, imaging

HeLa cells were cultured in a DMEM medium that contained 10% FPS at 37 °C in a 5% CO_2_ atmosphere. After incubating HeLa cells with NPs (20 µg/mL) in glass bottom dishes for 4 h, 200 nM Lyso-Tracker was added, incubated for 30 min. After that, the dishes were washed with PBS 3 times and visualized by Confocal laser scanning microscopy (CLSM) immediately.

### Molecule descriptors

Molecule descriptors were a crucial step in molecular machine learning to encode molecules and extract structural information. Quantitative structure-activity relationship (QSAR) was a crucial tool in chemometrics. It used mathematical-statistical methods to explain the relationship between a compound’s activity or physicochemical characteristics and its molecular structure. The foundation of QSAR studies was the calculation of molecular descriptors, and the precise definition and logical application of these descriptors were crucial to QSAR studies. The ability to obtain QSAR models with high confidence and validity depended mainly on the correct choice of descriptors. A molecular descriptor measured a molecule’s characteristics in a specific area, such as a physicochemical property or a numerical index derived from the molecule’s structure by different algorithms. More than 5000 molecular descriptors were currently accessible in a variety of software. RDKit was used to produce molecular descriptors as numerical descriptors for prediction experiments. There were two types of molecular descriptors: quantitative and qualitative. Quantitative descriptions were based on molecular graph theory, various theoretical or experimental spectral data (e.g., UV spectra), molecular composition (e.g., number of hydrogen bond donors, number of chemical bonds), physicochemical properties (e.g., ester water distribution coefficients) descriptors, molecular field descriptors, and molecular shape descriptors. Qualitative descriptors were generally referred to as molecular fingerprints. That is, some code represents a molecule’s structure, properties, fragments, or substructures. All molecular descriptors were generated by RDKit(http://www.rdkit.org).

#### Quantitative descriptors

Depending on the computational demands of the molecular structure dimension, quantitative descriptors could be categorized as one-dimensional, two-dimensional, three-dimensional, etc. To compute descriptors, RDKit offers a variety of methods that could be applied to molecular screening, drug toxicity testing, and other applications. Herein, 196 one- and two-dimensional descriptors, including 106 one- and 90 two-dimensional molecular descriptors, had been screened to quantify features.

#### Qualitative descriptors

Qualitative molecular descriptors were also known as molecular fingerprints. One of the most critical problems encountered when comparing similarities between two compounds was the complexity of the task. To make the comparison of molecules computationally easier, a certain degree of simplification or abstraction was required. A molecular fingerprint was an abstract representation of a molecule that converts (encodes) it into many bit strings (also known as bit vectors) that were then easily compared between molecules. A typical procedure extracted a molecule’s structural characteristics before hashing them to create the bit vector. Comparing molecules was hard, comparing bit vectors was easy, and comparisons between molecules must be quantifiable. Each bit on a molecular fingerprint corresponded to a molecule fragment. Molecular fingerprints were classified into several types based on the method used to convert the molecular representation into bit vectors. Common molecular fingerprinting methods include the morgan circular fingerprint, daylight fingerprint, topological torsion fingerprint, and atom-pair fingerprint.

Extended connectivity fingerprint (ECFP) was a circular topological fingerprints designed for molecular characterization, similarity search, and structure-activity modeling. Morgan connectivity fingerprint (MCP) were part of ECFP, derived from Morgan’s algorithm, and have became the industry standard method for circular molecular fingerprints, designed explicitly for constructive relationship studies. They were often used in ML as a benchmark for comparing the performance of new strategies. When used, MCP first sets a defined diameter – different diameters produced different fingerprints – then employed the Morgan search algorithm to look for all substructures in the molecule with that diameter. Finally, it hashed to obtain each substructure’s hash value, forming the corresponding fingerprint. ECFPs with small diameters were typically appropriate for similarity searches and molecular clustering. Contrarily, ECFPs with large diameters gained from having more molecular structure information and were thus perfect for ML to make activity predictions.

Topological or path-based fingerprint started from an atom and took each substructure along the path until it reached a specified length, then hashed each substructure to obtain a molecular fingerprint. This fingerprint could be adjusted for quick substructure searching and molecular filtering and applied to any molecule. The most well-known examples of this type of fingerprint were daylight fingerprint, which had bits that could be up to 2048 bits long and encode every possible linkage pathway that a molecule could take to reach a specific length. Atom-pair fingerprint identified each molecule atom as the shortest path based on its environment. Topological torsion fingerprints were generated by constructing a topological double-angle descriptor using four non-hydrogen atom-pair bonding paths. Both fingerprints could be expressed in sparse form.

### Machine learning model

#### Random Forest (RF)

RF was a general-purpose ensemble learning algorithm that used the Classification and Regression Tree (CART) algorithm to reach the final conclusion after “aggregating” the results of a single fully grown regression tree constructed on a randomly chosen subset of data. Each regression tree selected a variable to reduce the Gini impurity as it grows

1 $$\begin{array}{c}{I}_{G}\left(p\right)=1-\sum _{i=1}^{J}{p}_{i}^{2}\end{array}$$

to lessen the chance that a new random variable would be incorrectly classified. In this case, J was the total number of classes, and pi was the likelihood that a given item belongs to class i. For the overall algorithm to be more predictive than a single regression tree and more resilient on a noisy database, RF uses bootstrap sampling and random selection of input samples to ensure that each regression tree in the forest was distinct and uncorrelated to one another. The algorithm’s accuracy would increase with a large number of regression trees.

#### Gradient boosting regression tree (GBRT)

The GBRT was a well-liked model that performed exceptionally well in ML applications. It was a boosting family representative algorithm. Boosting was a progressive model combination strategy. Each new regressor enhanced the predictions of the previous regressor. Thus, boosting was a technique for combining models that reduced bias. GBRT was an iterative regression tree algorithm that consisted of multiple trees. The integration technique used was gradient boosting, and the final result was the sum of the conclusions from each tree. The intuitive understanding was that each round of prediction has residuals with the actual values, the next round of prediction was made based on the residuals, and the result was obtained by summing all predictions. The GBRT process involved several iterations, with each iteration producing a weak regressor that was trained using the residuals of the previous regressor. Since the training process was made to reduce residuals, the accuracy of the final regressor was continually improved. Generally, the requirements for weak regressors were straightforward, with low variance and high bias. Classification and Regression Tree (CART) was usually chosen with weak regressors. The depth of each CART was limited due to the high bias and simplicity requirements. The final total regressor was a weighted average of the weak regressors from each training round. GBRT could be expressed as follows when a regression tree represents the basic model:

2 $$\begin{array}{c}{f}_{M}\left(X\right)=\sum _{m=1}^{M}T\left(X;{{\Theta }}_{m}\right)\end{array}$$

where $T\left(X;{{\Theta }}_{m)}\right)$ represents the regression tree. M was the number of trees. The forward distribution algorithm was adopted first to determine the initial boosting tree ${f}_{0}\left(X\right)=0$. Then the model in step m was:

3 $$\begin{array}{c}{\widehat{{\Theta }}}_{m}=arg\underset{{{\Theta }}_{m}}{\text{min}}\sum _{i=1}^{N}L({y}_{i},{f}_{\left(m-1\right)}\left({X}_{i}\right)+T({X}_{i};{{\Theta }}_{m}))\end{array}$$

where the loss function L() was used, the mean square error and the absolute value error were typically the loss functions chosen by the regression algorithm.

#### K-nearest neighbor (KNN)

KNN was one of the most basic regression algorithms. When the k-nearest samples of a data point were considered, the value of that data point was the average of those k values. The number of neighbors k and the calculation of distance were two crucial factors influencing KNN. K was usually an integer no larger than 20, and distance was calculated using the Euclidean distance. Euclidean distance was defined as

4 $$\begin{array}{c}d=\sqrt{{\sum }_{i=0}^{n}{\left({x}_{i}-{y}_{i}\right)}^{2}}\end{array}$$

Where n was the number of samples.

#### Support vector machine (SVM)

The Vapnik-Chervonenkis theory was the basis for the development of SVM, also known as “support vector network,“ which was a kernel-based supervised learning algorithm. For regression issues, the SVM calculated a hyperplane and fit training data to the hyperplane using a kernel function to project input data onto a higher dimensional space. The kernel function for this work was linear.

#### Extreme gradient boosting (XGBoost)

The XGBoost algorithm was an upgraded library of the GBRT algorithm, which significantly increased data processing effectiveness and lowered the risk of overfitting. Because it employed a sparse-aware algorithm for sparse data and trained the weighting function using first- and second-order derivatives, it was more scalable than GBRT. Similar to GBRT, XGBoost also employed a forward stepwise algorithm, and XGBoost chose the parameters for the following decision tree by minimizing structural risk.

5 $$\begin{array}{c}{\widehat{{\Theta }}}_{m}=arg\underset{{{\Theta }}_{m}}{\text{min}}\sum _{i=1}^{N}L({y}_{i},{f}_{\left(m-1\right)}\left({X}_{i}\right)+{\Omega }({X}_{i};{{\Theta }}_{m}))\end{array}$$

where ${\Omega }({X}_{i};{{\Theta }}_{m})$ represented the regularisation term of the regression tree, which was an important difference between XGBoost and GBRT. Similar hyperparameters were used by XGBoost and GBRT.

#### Multilayer perceptron (MLP)

MLP was a forward-structured artificial neural network that mapped a set of input vectors to a set of output vectors. The backpropagation algorithm, a supervised learning technique, was frequently used to train MLPs, which mimicked the human nervous system’s learning and data prediction processes. It first learned, then stored the data with weights and employed algorithms to modify the weights and lessen bias in the training process or the difference between the actual and predicted values. The input, hidden, and output layers were the three types of network layers that made up an MLP. Each layer was made up of a specific number of nodes, which were neurons with non-linear activation functions. Each layer was fully connected to the one before it. The input layer was used to receive data, the hidden layer was used to process the data, and the output layer offered the final prediction. A single network layer’s output was depicted as

6 $$\begin{array}{c}f\left(x\right)=f\left(\sum _{i}^{M}{\omega }_{i}{x}_{i}+b\right)\end{array}$$

where x represented the input to the node, w represented the node’s weight, b represented the bias, and f(x) represented the activation function. If each neuron’s activation function was linear, an MLP with multiple layers could be compared to a single-layer neural network. Rectified linear unit (ReLU) was a non-linear activation function used in this work.

#### Convolution neural network (CNN)

The convolutional neural network was a feed-forward neural network with artificial neurons that responded to a portion of the surrounding units in the coverage area. CNN comprised three layers: the input layer, the hidden layer, and the output layer, with the hidden layer containing various types of networks such as convolutional, pooling, fully connected (similar to classical neural networks), and normalization layers. The convolutional layer was the core of the CNN and performed the dot product of the convolutional kernel and the layer input matrix, this product was usually the Frobenius inner product, and the activation function was ReLU. The convolution operation produced a feature map as the convolutional kernel moved along the layer’s input matrix. This feature map then became part of the input for the subsequent layer. CNN was a desirable deep learning structure because it required fewer parameters to be considered than other deep neural networks.

### Metrics

***MAE (mean absolute error)*** of these n samples was given by

7 $$\begin{array}{c}MAE=\frac{1}{n}\sum _{i=1}^{n}\left|{y}_{true}^{\left(i\right)}-{y}_{pred}^{\left(i\right)}\right|\end{array}$$

***RMSE (root mean squared error)*** of these n samples was given by

8 $$\begin{array}{c}RMSE=\sqrt{\frac{1}{n}\sum _{i=1}^{n}{\left({y}_{true}^{\left(i\right)}-{y}_{pred}^{\left(i\right)}\right)}^{2}}\end{array}$$

***Coefficient of determination (R2)*** of these n samples was given by

9 $$\begin{array}{c}{R}^{2}=1-\frac{\sum _{i=1}^{n}{({y}_{true}^{\left(i\right)}-{y}_{pred}^{\left(i\right)})}^{2}}{\sum _{i=1}^{n}{({y}_{true}^{\left(i\right)}-\frac{1}{n}{\sum }_{j=1}^{n}{y}_{true}^{\left(j\right)})}^{2}}\end{array}$$

### Hyperparameters

We employed Bayesian optimization to identify each model’s ideal hyperparameters during model training[54]. This step was crucial because it has been demonstrated that properly tuned hyperparameters could produce predictions with better accuracy than those selected by hand.

### 10-fold cross-validation

The data were randomly divided into ten equally sized mutually exclusive subsets, each keeping the data distribution as consistent as possible. Nine subsets were taken at a time for the training set and one for the test set. This yielded ten training and test sets, and the final result was the mean of the outcomes of the ten tests.

## Electronic supplementary material

Below is the link to the electronic supplementary material.

Scheme S1. The synthetic route to prepare PTMM, TTNA, and TTBI.
Figure S1 ^1^ H NMR spectrum of PM.
Figure S2 ^1^ H NMR spectrum of PTMM.
Figure S3 ^13^ C NMR spectrum of PTMM.
Figure S4. LC-MS spectrum of PTMM.
Figure S5 ^1^ H NMR spectrum of TTA.
Figure S6 ^1^ H NMR spectrum of TTNA.
Figure S7 ^13^ C NMR spectrum of TTNA.
Figure S8. MALDI-TOF-MS spectrum of TTNA.
Figure S9 ^1^ H NMR spectrum of TTBI.
Figure S10. ^13^ C NMR spectrum of TTBI.
Figure S11. LC-MS spectrum of TTBI.
Figure S12. The absorption spectrum of AIEgens in different solvents.
Figure S13. PL spectra of AIEgens of AIEgens with different water fractions.
Table S1. Comparison of absorption and emission peak between experimental and ML predicted.
Table S2. Particle size of AIEgens NPs.
Table S3. Zeta potentials of AIEgens NPs.
Figure S14. Calculated LUMO and HOMO of PTMM, TTNA, and TTBI.
Figure S15. Z-stack images of phantom of PTMM NPs.
Figure S16. Z-stack images of phantom of TTNA NPs.
Figure S17. Z-stack images of phantom of TTBI NPs.
Figure S18. Experimental and predicted data are compared using 10-fold cross-validation.
Figure S19. ML prediction error distribution.
Figure S20. Model scalability.
Figure S21. Comparison of ML accuracy and TD-DFT.
Figure S22. Illustration of 10-fold cross-validation.

## Data Availability

All data generated or analysed during this study are included in this published article and its additional file.
