# Supplementary material for: Machine-learning screening of luminogens with aggregation-induced emission characteristics for fluorescence imaging
Source: J Nanobiotechnology. 2023 Mar 25;21:107. doi: 10.1186/s12951-023-01864-9 (PMC10039567; doi:10.1186/s12951-023-01864-9)
Supplement: Supplementary file 1 — Scheme S1. The synthetic route to prepare PTMM, TTNA, and TTBI. Figure S1 1 H NMR spectrum of PM. Figure S2 1 H NMR spectrum of PTMM. Figure S3 13 C NMR spectrum of PTMM. Figure S4. LC-MS spectrum of PTMM. Figure S5 1 H NMR spectrum of TTA. Figure S6 1 H NMR spectrum of TTNA. Figure S7 13 C NMR spectrum of TTNA. Figure S8. MALDI-TOF-MS spectrum of TTNA. Figure S9 1 H NMR spectrum of TTBI. Figure S10. 13 C NMR spectrum of TTBI. Figure S11. LC-MS spectrum of TTBI. Figure S12. The absorption spectrum of AIEgens in different solvents. Figure S13. PL spectra of AIEgens of AIEgens with different water fractions. Table S1. Comparison of absorption and emission peak between experimental and ML predicted. Table S2. Particle size of AIEgens NPs. Table S3. Zeta potentials of AIEgens NPs. Figure S14. Calculated LUMO and HOMO of PTMM, TTNA, and TTBI. Figure S15. Z-stack images of phantom of PTMM NPs. Figure S16. Z-stack images of phantom of TTNA NPs. Figure S17. Z-stack images of phantom of TTBI NPs. Figure S18. Experimental and predicted data are compared using 10-fold cross-validation. Figure S19. ML prediction error distribution. Figure S20. Model scalability. Figure S21. Comparison of ML accuracy and TD-DFT. Figure S22. Illustration of 10-fold cross-validation. [file 12951_2023_1864_MOESM1_ESM.docx]

# Supporting Information

# Machine-Learning Screening of Luminogens with Aggregation-Induced Emission Characteristics for Fluorescence Imaging

Yibin Zhang^a^, Miaozhuang Fan^a^, Zhourui Xu^a^, Yihang Jiang^a^, Huijun Ding^a^, Zhengzheng Li^a^, Kaixin Shu^a^, Mingyan Zhao^a^, Gang Feng^a^, Ken-Tye Yong^c^, Biqin Dong^d^, Wei Zhu^b*^, Gaixia Xu^a*^

1. Guangdong Key Laboratory for Biomedical Measurements and Ultrasound Imaging, School of Biomedical Engineering, Health Science Center, Shenzhen University, Shenzhen, Guangdong 518055, China
2. Key Laboratory of Advanced Textile Materials and Manufacturing Technology and Engineering Research Center for Eco-Dyeing & Finishing of Textiles, Ministry of Education, Zhejiang Provincial Engineering Research Center for Green and Low-carbon Dyeing & Finishing, Zhejiang Sci-Tech University, Hangzhou 310018, China
3. School of Biomedical Engineering, The University of Sydney, Sydney, New South Wales, 2006, Australia
4. Guangdong Provincial Key Laboratory of Durability for Marine Civil Engineering, College of Civil and Transportation Engineering, Shenzhen University, Shenzhen, 518060, China

Corresponding author: Wei Zhu, [willian_fox@zstu.edu.cn](mailto:willian_fox@zstu.edu.cn); Gaixia Xu, [xugaixia@szu.edu.cn](mailto:xugaixia@szu.edu.cn)

**Table of Contents**

**Scheme S1.** The synthetic route to prepare PTMM, TTNA, and TTBI.

**Figure S1.** ^1^H NMR spectrum of PM.

**Figure S2.** ^1^H NMR spectrum of PTMM.

**Figure S3.** ^13^C NMR spectrum of PTMM.

**Figure S4.** LC-MS spectrum of PTMM.

**Figure S5.** ^1^H NMR spectrum of TTA.

**Figure S6.** ^1^H NMR spectrum of TTNA.

**Figure S7.** ^13^C NMR spectrum of TTNA.

**Figure S8.** MALDI-TOF-MS spectrum of TTNA.

**Figure S9.** ^1^H NMR spectrum of TTBI.

**Figure S10.** ^13^C NMR spectrum of TTBI.

**Figure S11.** LC-MS spectrum of TTBI.

**Figure S12.** The absorption spectrum of AIEgens in different solvents.

**Figure S13.** PL spectra of AIEgens of AIEgens with different water fractions.

**Table S1.** Comparison of absorption and emission peak between experimental and ML predicted.

**Table S2.** Particle size of AIEgens NPs.

**Table S3.** Zeta potentials of AIEgens NPs.

**Figure S14.** Calculated LUMO and HOMO of PTMM, TTNA, and TTBI.

**Figure S15.** Z-stack images of phantom of PTMM NPs.

**Figure S16.** Z-stack images of phantom of TTNA NPs.

**Figure S17.** Z-stack images of phantom of TTBI NPs.

**Figure S18.** Experimental and predicted data are compared using 10-fold cross-validation.

**Figure S19.** ML prediction error distribution.

**Figure S20.** Model scalability.

**Figure S21.** Comparison of ML accuracy and TD-DFT.

**Figure S22.** Illustration of 10-fold cross-validation.

**Synthesis of PTMM, TTNA, and TTBI**

Scheme S1. Synthetic routes to PTMM, TTNA, and TTBI


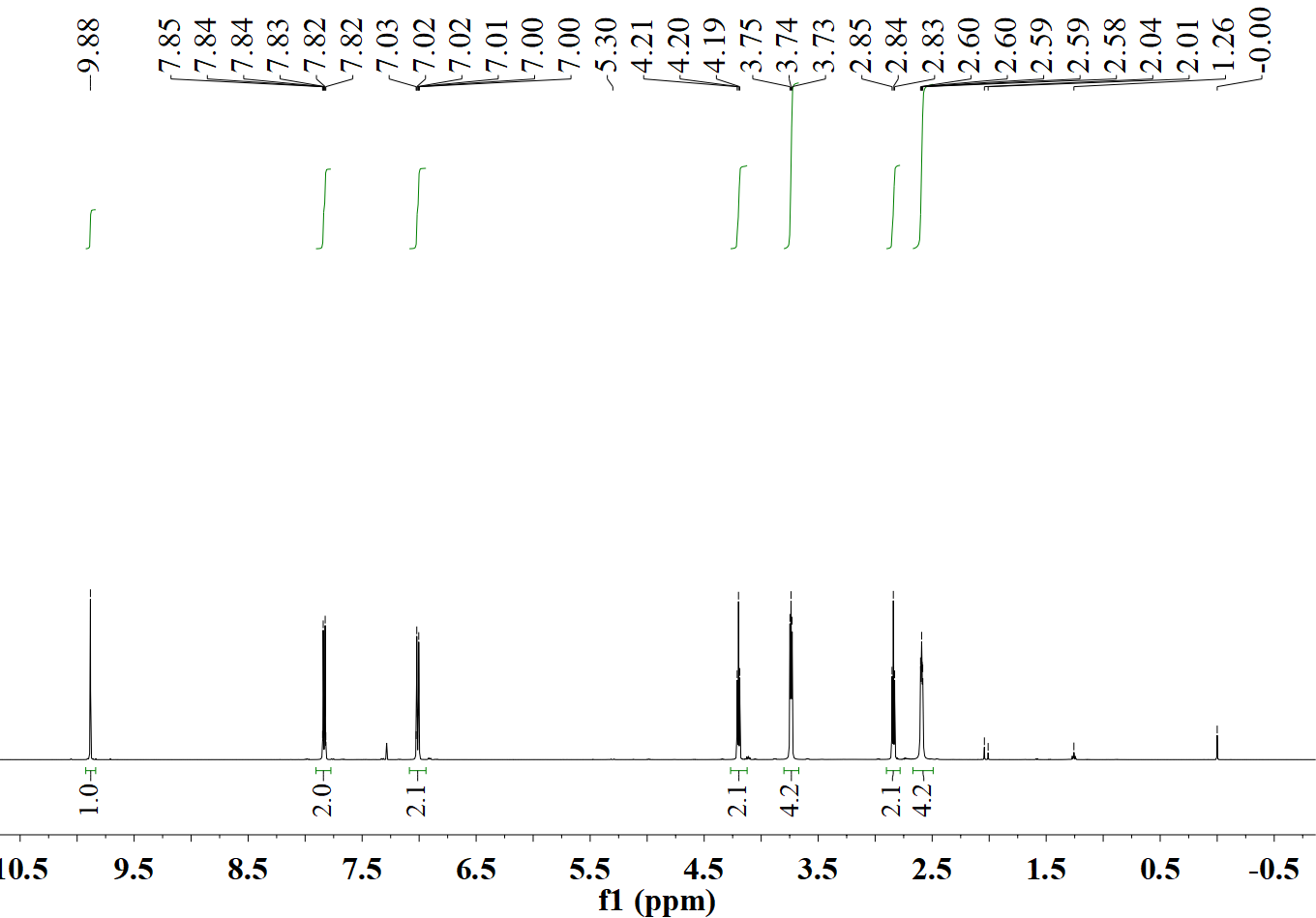


Figure S1. ^1^H NMR spectrum of PM


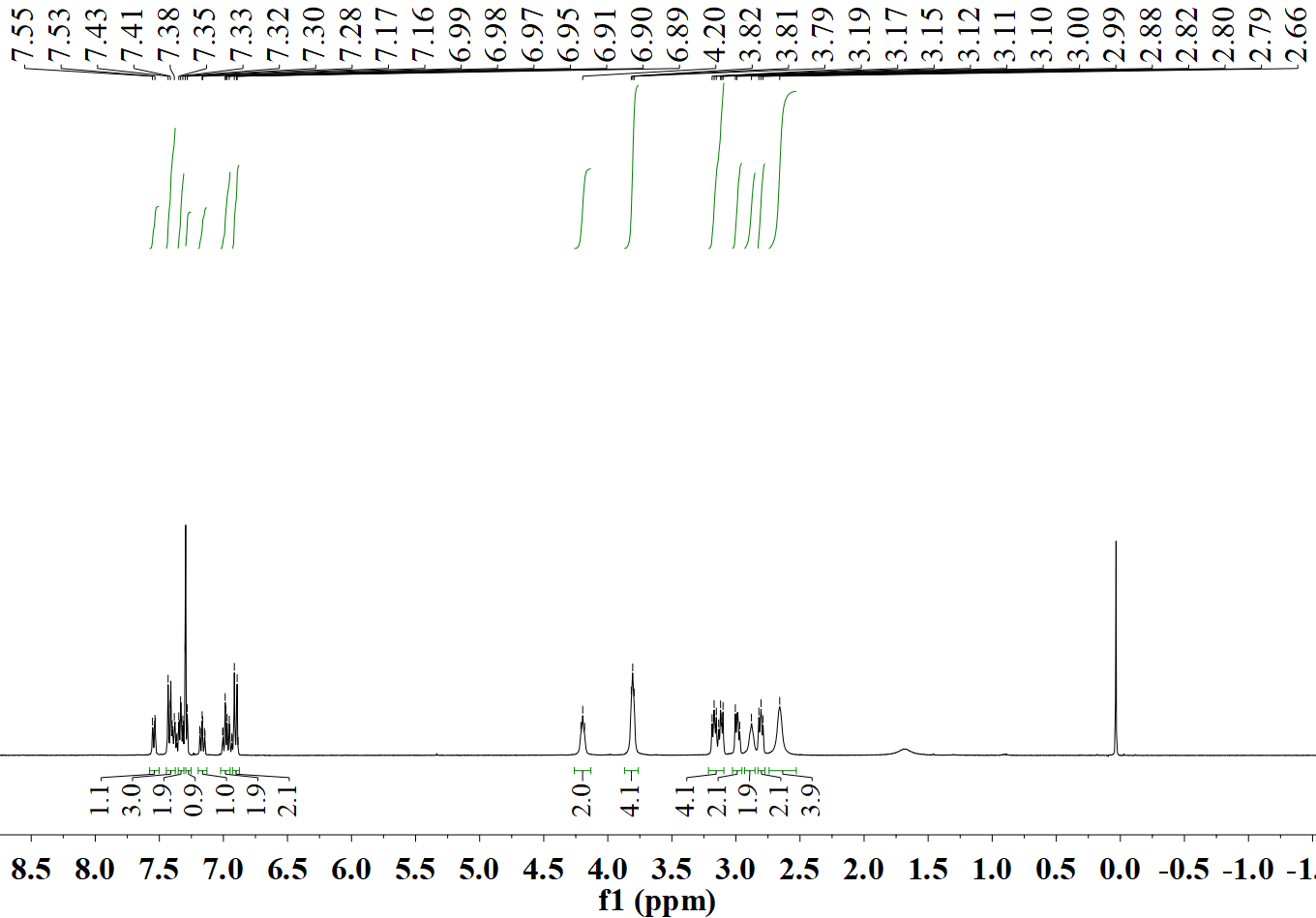


Figure S2. ^1^H NMR spectrum of PTMM


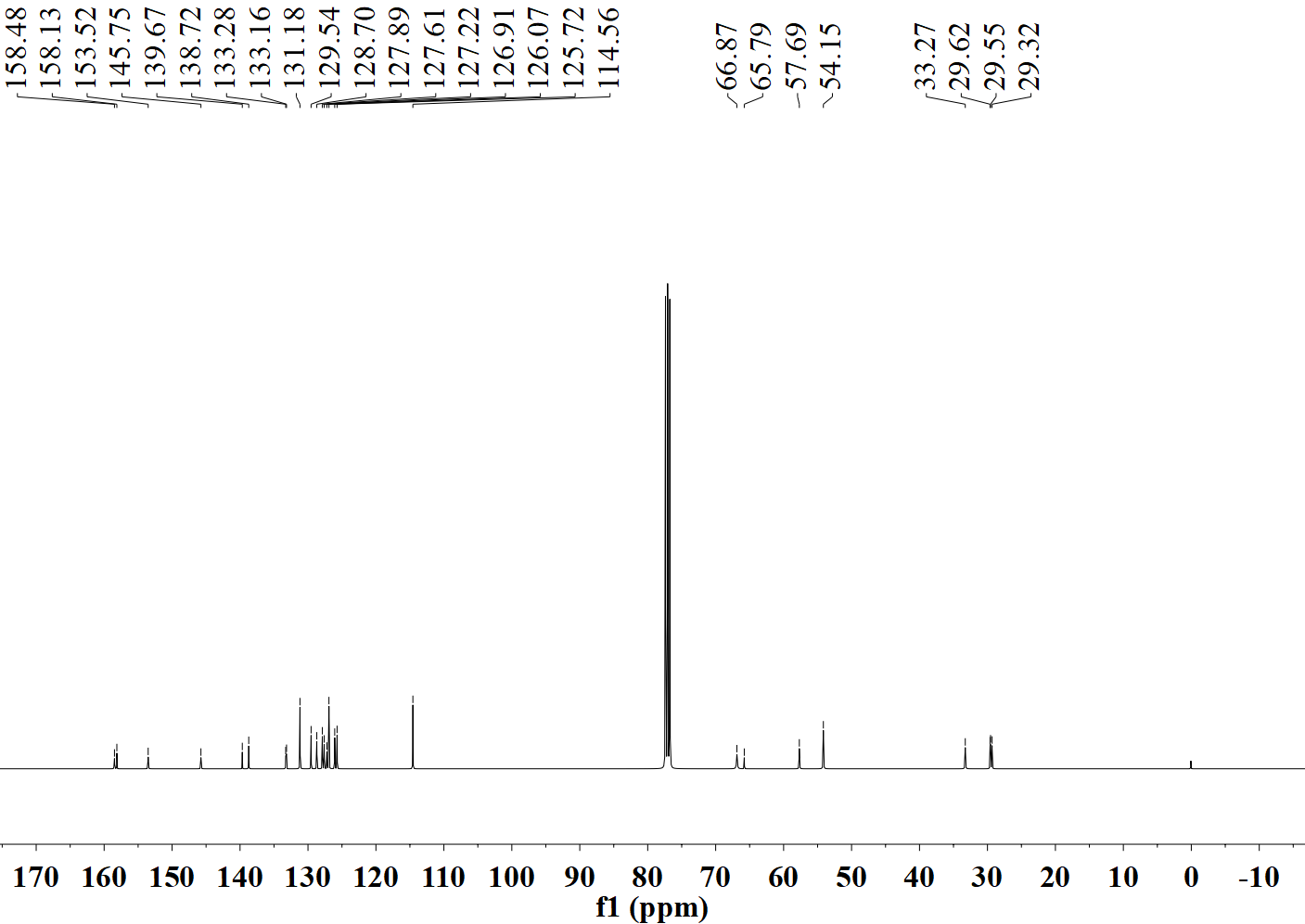


Figure S3. ^13^C NMR spectrum of PTMM


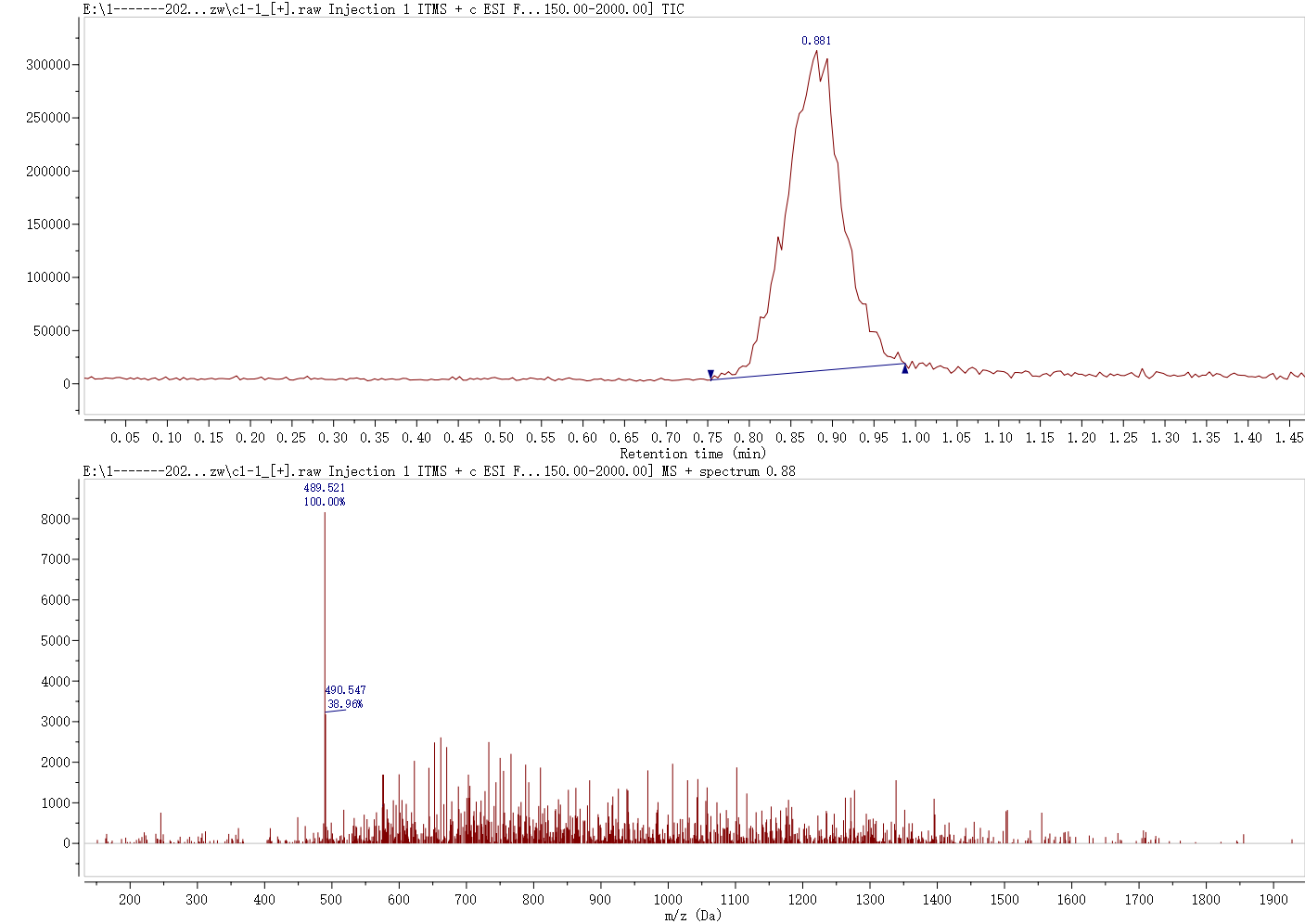


Figure S4. LC-MS spectrum of PTMM


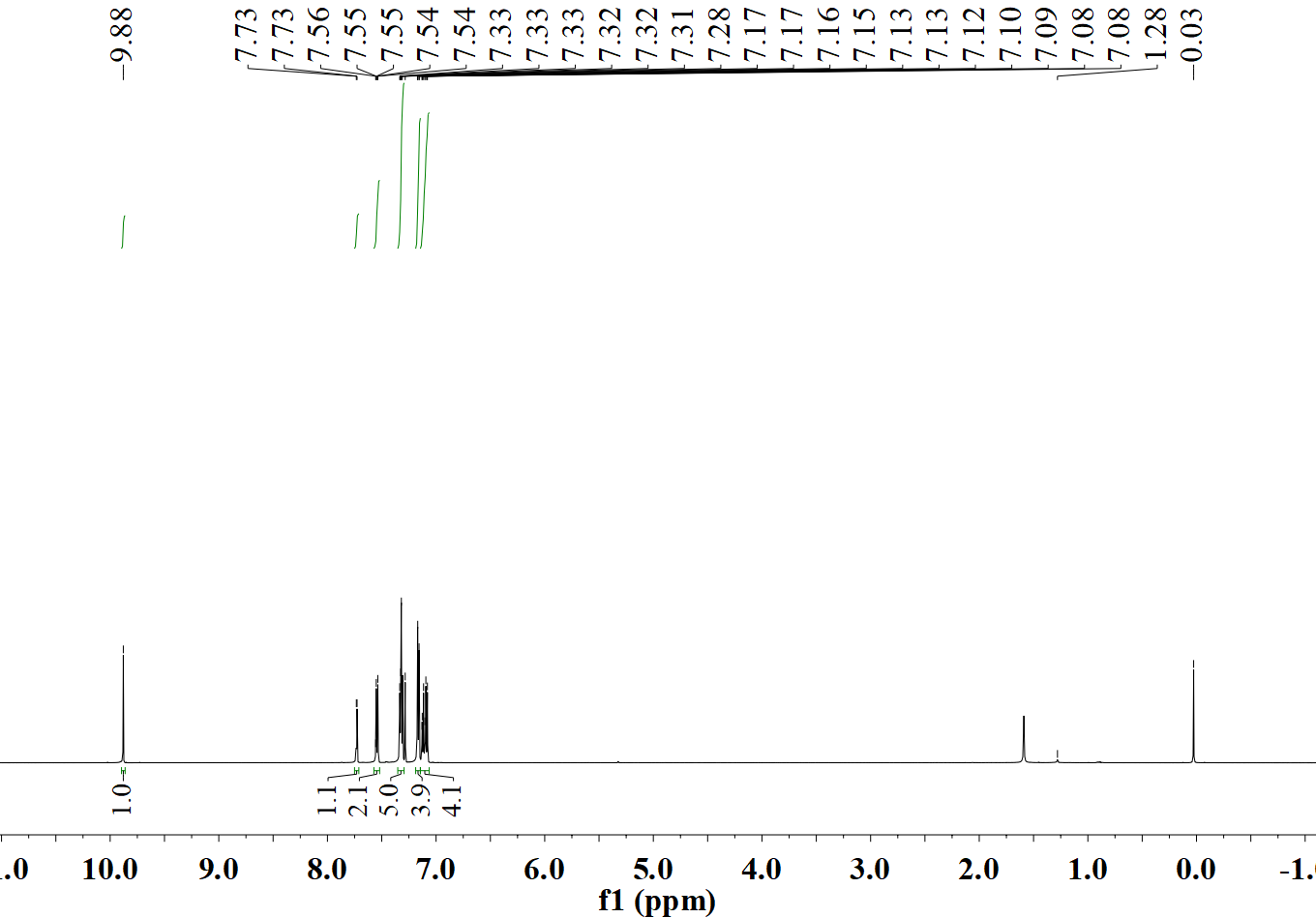


Figure S5. ^1^H NMR spectrum of TTA


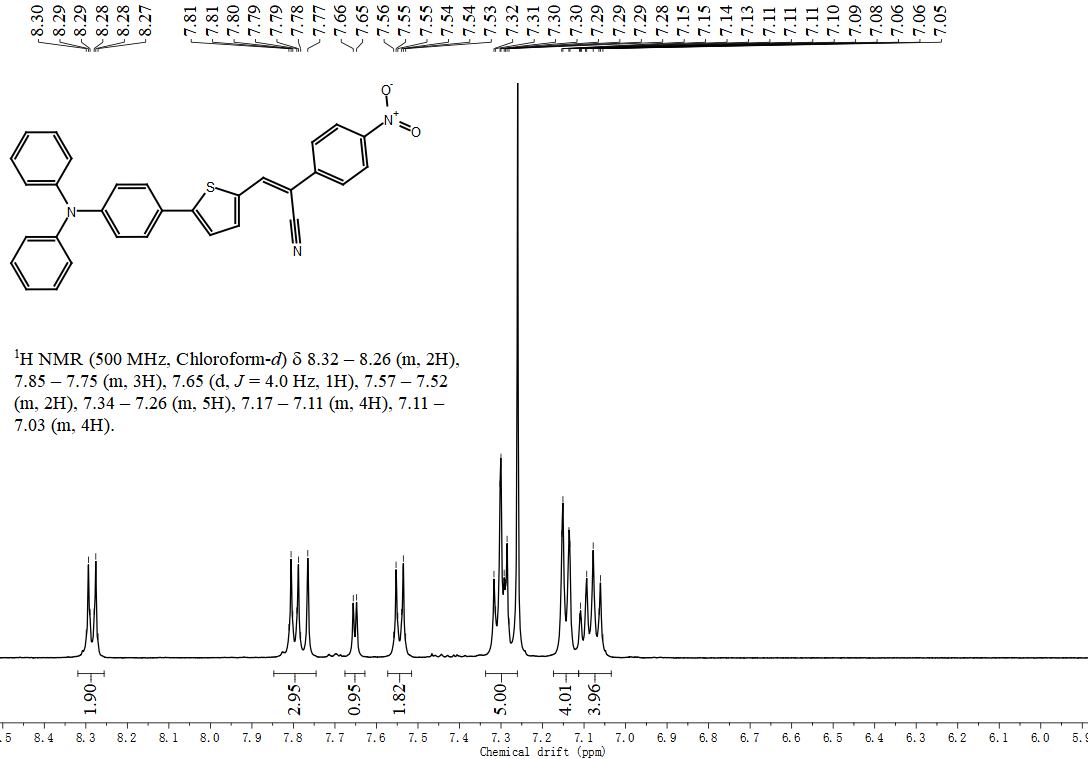


Figure S6. ^1^H NMR spectrum of TTNA


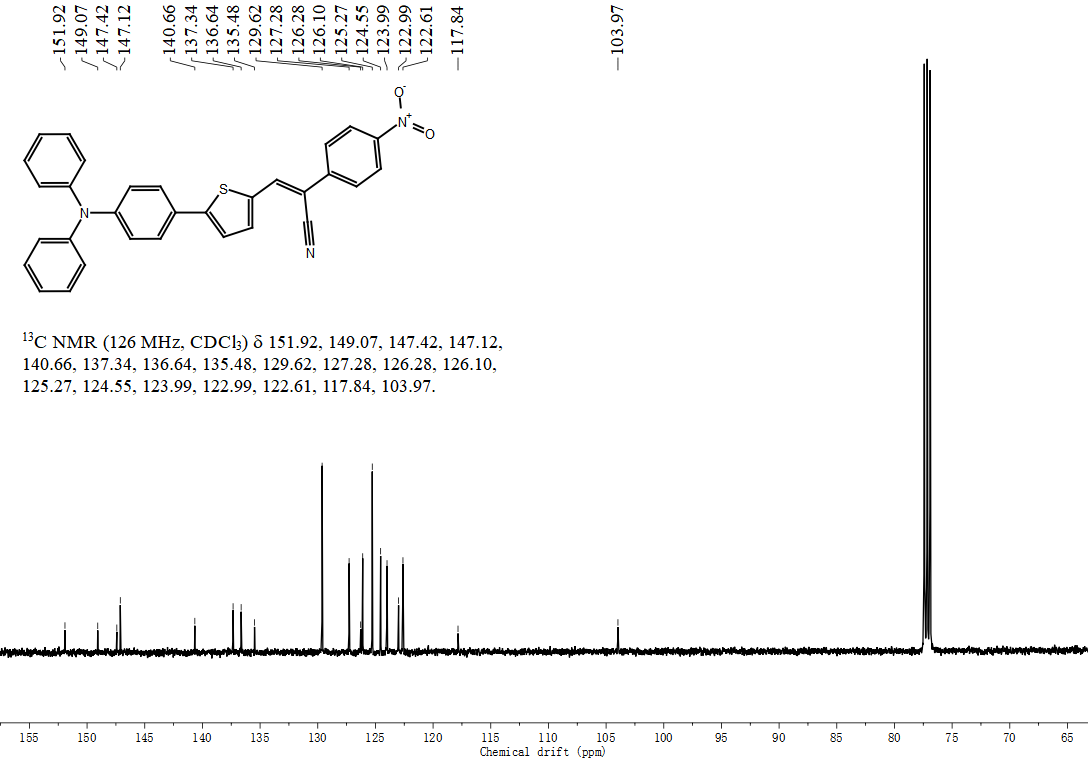


Figure S7. ^13^C NMR spectrum of TTNA

Figure S8. MALDI-TOF-MS spectrum of TTNA


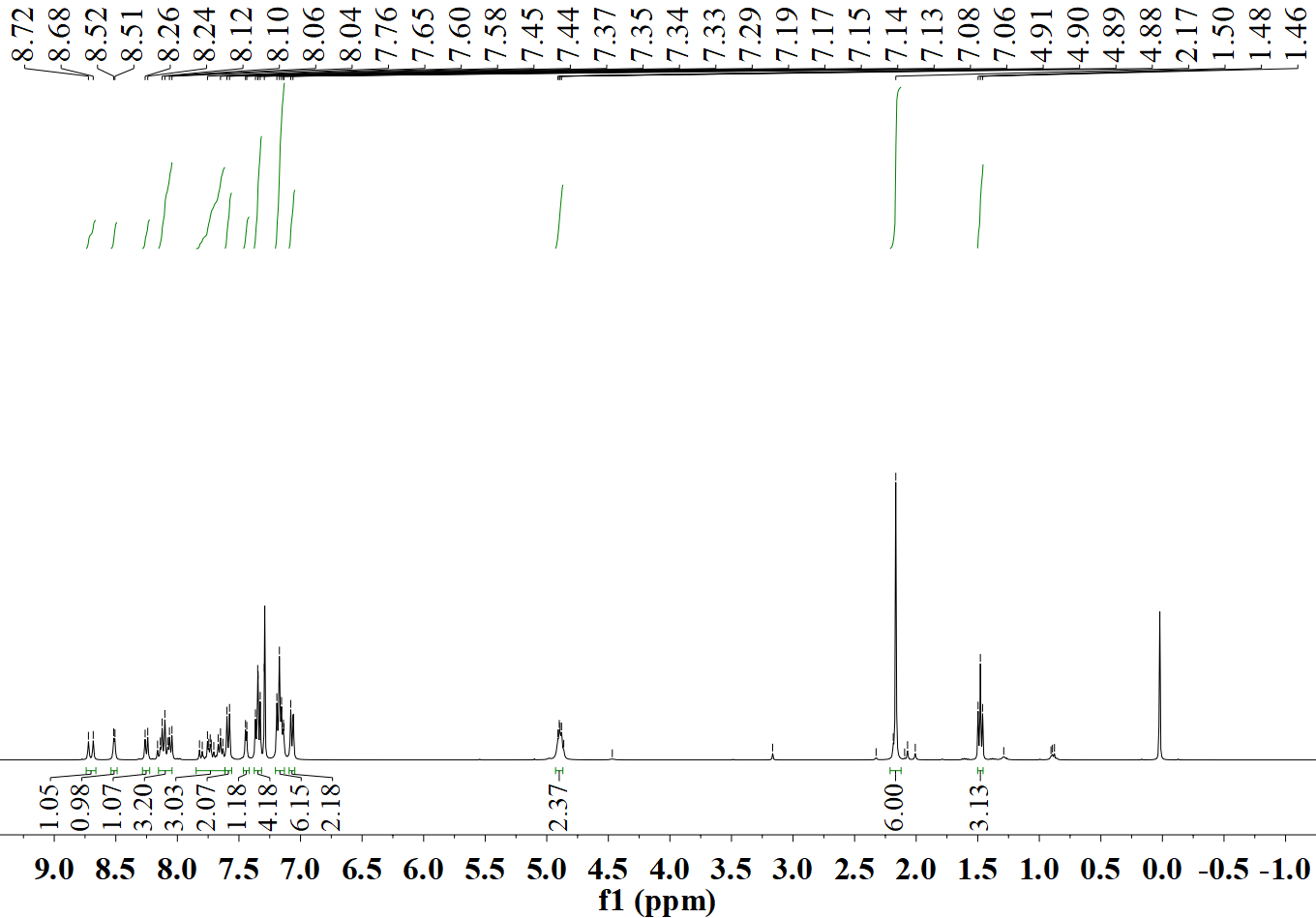


Figure S9. ^1^H NMR spectrum of TTBI


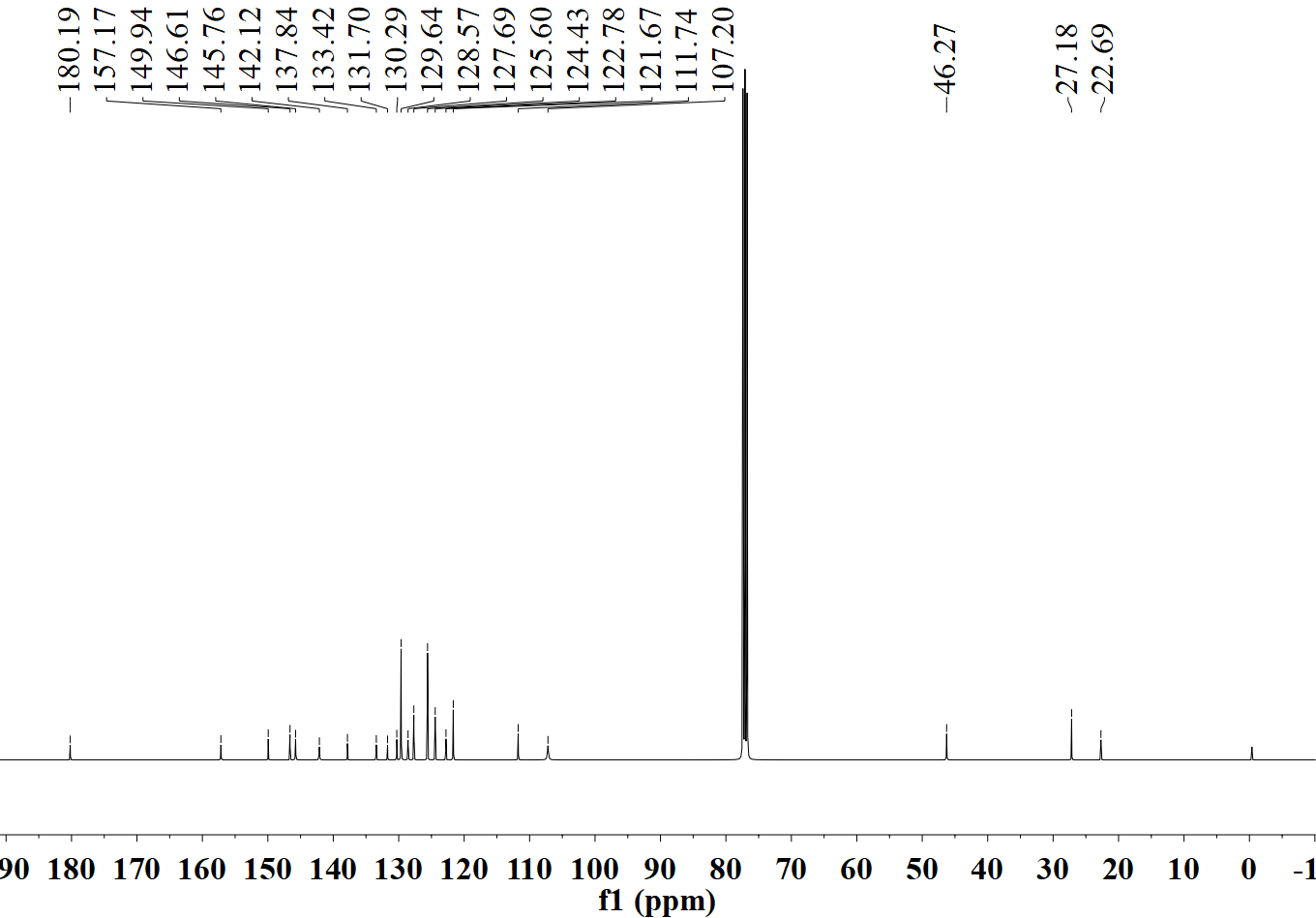


Figure S10. ^13^C NMR spectrum of TTBI


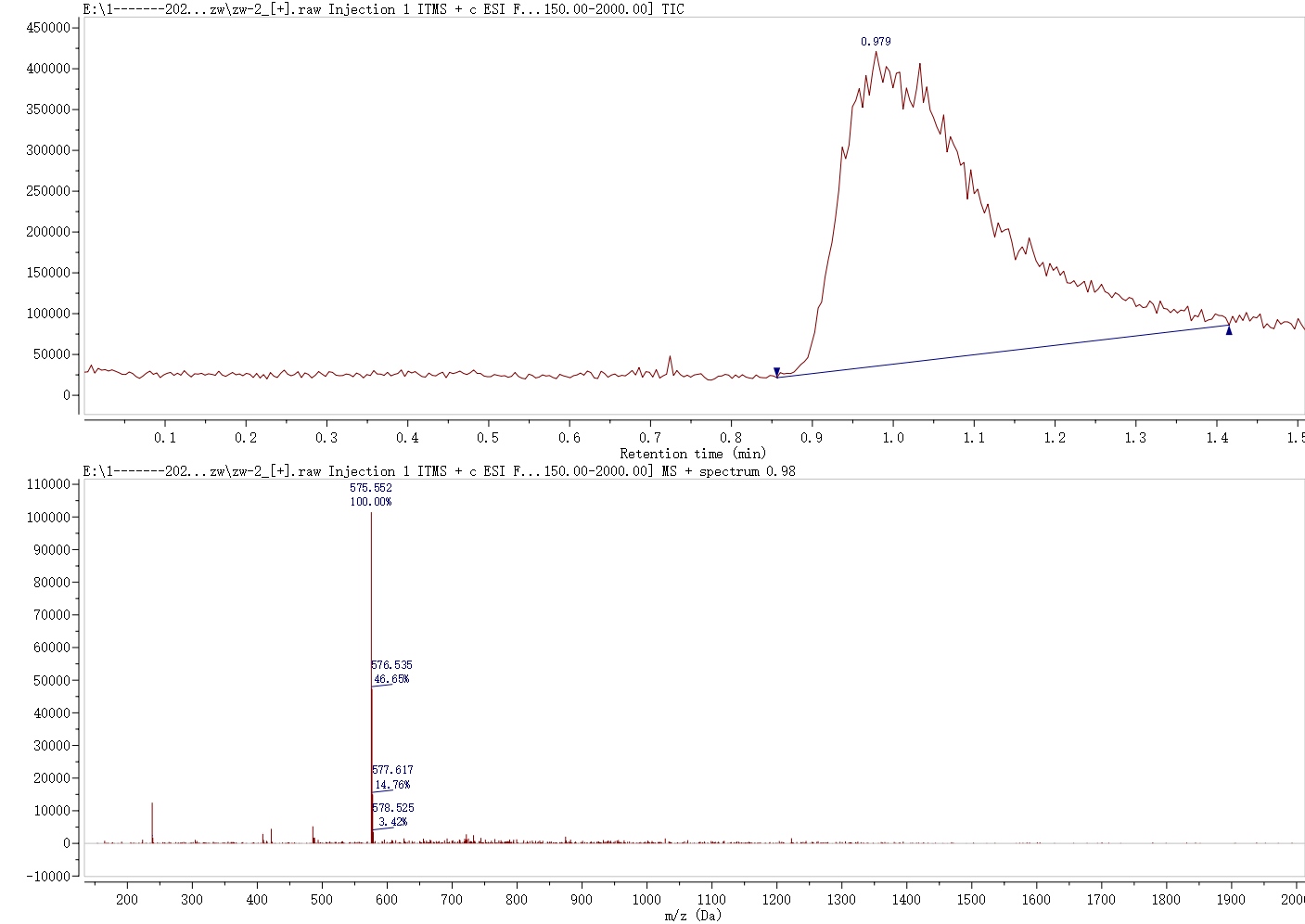


Figure S11. LC-MS spectrum of TTBI


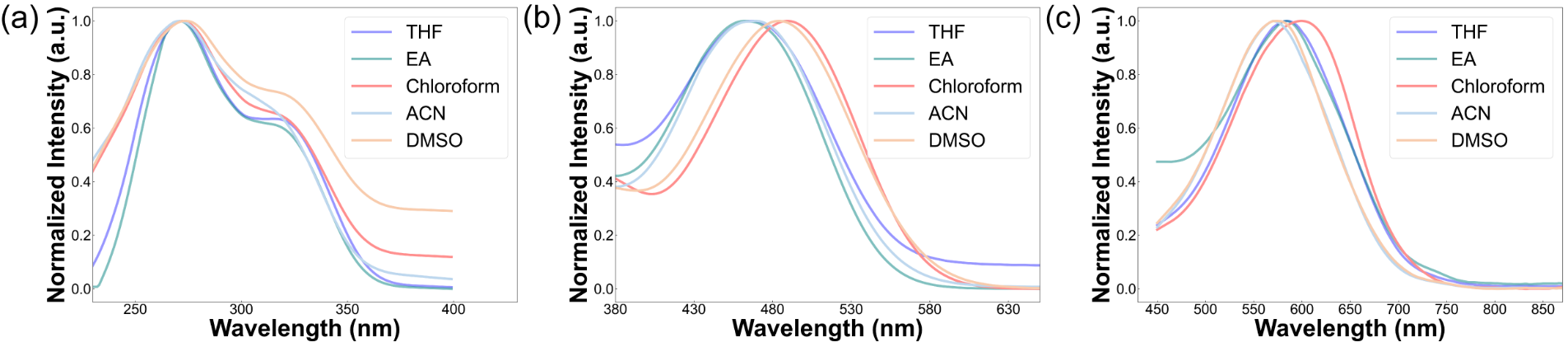


Figure S12. The normalized absorption spectrum in various solvents of (a) PTMM, (b) TTNA, and (c) TTBI.


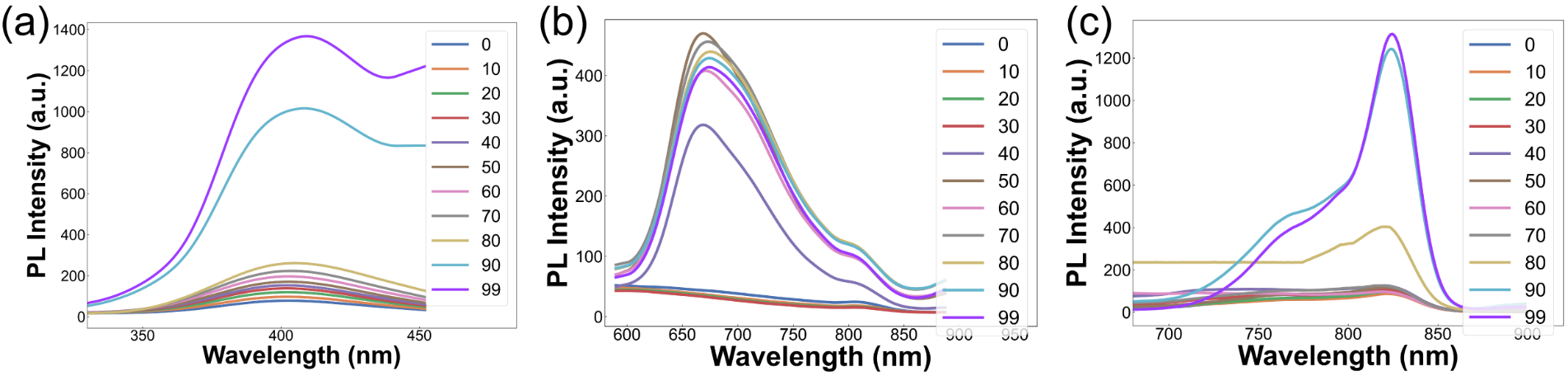


Figure S13. PL spectra of AIEgens with different water fractions. (a) PTMM, (b) TTNA, and (c) TTBI.

Table S1. Comparison of the absorption and emission peak between experimental and ML predicted

|  | THF | | EA | | Chloroform | | ACN | | DMSO | |
| --- | --- | --- | --- | --- | --- | --- | --- | --- | --- | --- |
|  | λ_abs_ | λ_em_ | λ_abs_ | λ_em_ | λ_abs_ | λ_em_ | λ_abs_ | λ_em_ | λ_abs_ | λ_em_ |
| PTMM |  |  |  |  |  |  |  |  |  |  |
| ML Predicted | 281 | 406 | 280 | 406 | 293 | 411 | 281 | 413 | 277 | 413 |
| Experimental | 268 | 400 | 267 | 402 | 269 | 409 | 269 | 405 | 266 | 402 |
| TTNA |  |  |  |  |  |  |  |  |  |  |
| ML Predicted | 487 | 672 | 478 | 667 | 502 | 666 | 491 | 661 | 483 | 667 |
| Experimental | 473 | 658 | 466 | 647 | 490 | 663 | 483 | 655 | 469 | 684 |
| TTBI |  |  |  |  |  |  |  |  |  |  |
| ML Predicted | 580 | 812 | 563 | 809 | 605 | 804 | 564 | 815 | 564 | 818 |
| Experimental | 583 | 827 | 583 | 820 | 600 | 816 | 580 | 821 | 578 | 828 |

Table S2. Particle size of AIEgens NPs

|  | PTMM NPs (nm) | TTNA NPs (nm) | TTBI NPs (nm) |
| --- | --- | --- | --- |
| Day 1 | 119.67±5.1 | 89.46±4.6 | 104.29±6.3 |
| Day 2 | 123.32±4.6 | 92.39±3.1 | 102.31±3.2 |
| Day 3 | 120.06±7.2 | 90.13±5.2 | 99.62±2.6 |
| Day 4 | 124.42±5.1 | 87.43±4.7 | 101.36±5.9 |
| Day 5 | 119.36±6.4 | 91.26±6.4 | 97.54±6.4 |
| Day 6 | 120.73±7.9 | 93.17±8.6 | 105.28±7.8 |
| Day 7 | 118.79±8.2 | 92.69±7.3 | 103.72±9.3 |

Table S3. Zeta potentials of AIEgens NPs

|  | PTMM NPs (mV) | TTNA NPs (mV) | TTBI NPs (mV) |
| --- | --- | --- | --- |
| Day 1 | -25.9±2.3 | -22.6±1.6 | -19.3±2.1 |
| Day 2 | -26.6±3.1 | -24.3±2.6 | -17.6±1.6 |
| Day 3 | -24.1±3.6 | -21.9±1.2 | -18.9±2.3 |
| Day 4 | -22.4±2.6 | -20.2±3.2 | -20.1±3.2 |
| Day 5 | -25.2±3.8 | -22.3±2.7 | -16.9±4.1 |
| Day 6 | -26.3±2.9 | -23.7±1.9 | -18.5±3.7 |
| Day 7 | -23.9±3.2 | -21.1±2.8 | -19.8±2.3 |


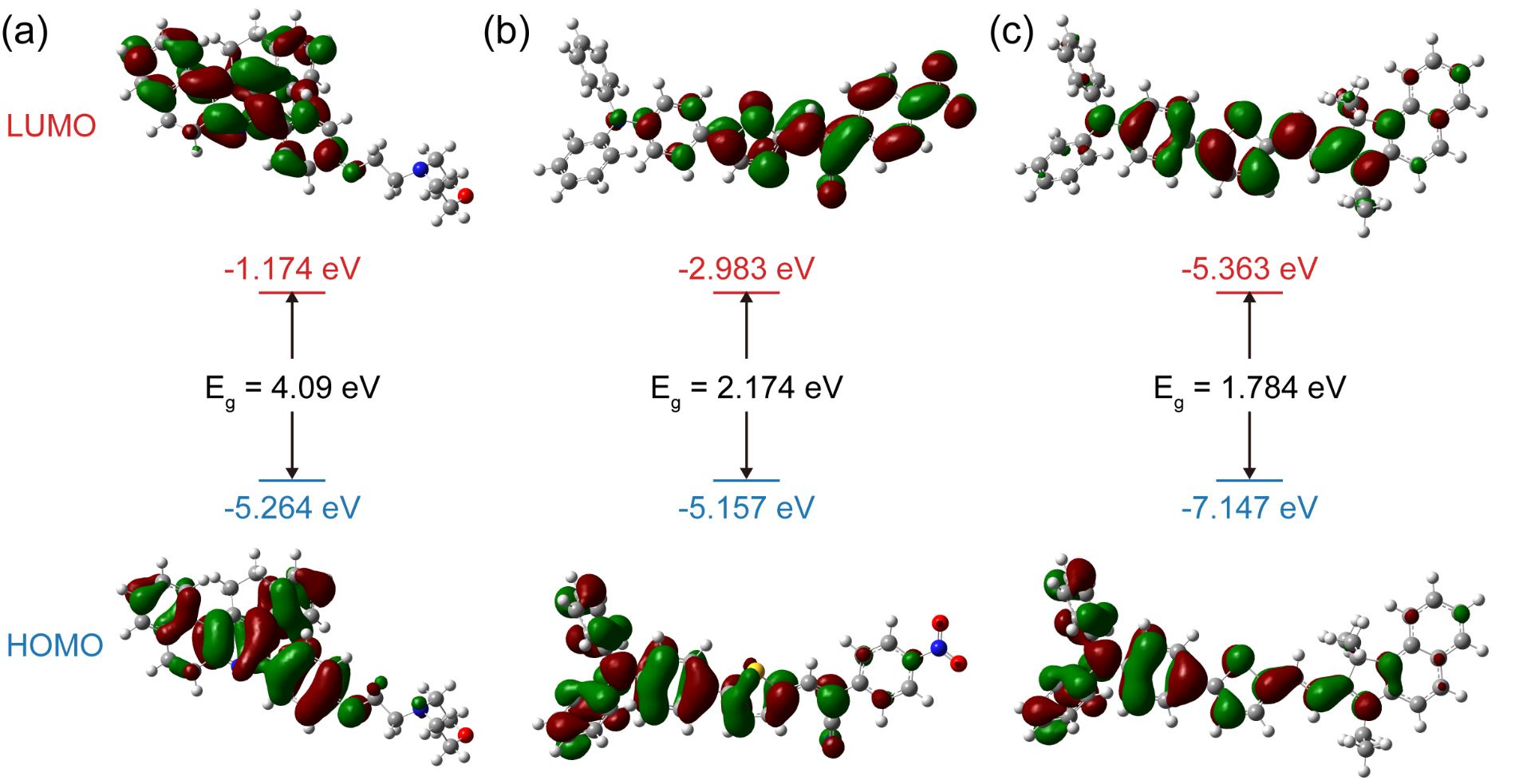


Figure S14. Calculated LUMO and HOMO of (a) PTMM, (b) TTNA, and (c) TTBI. E_g_ (energy gap)=LUMO-HOMO.


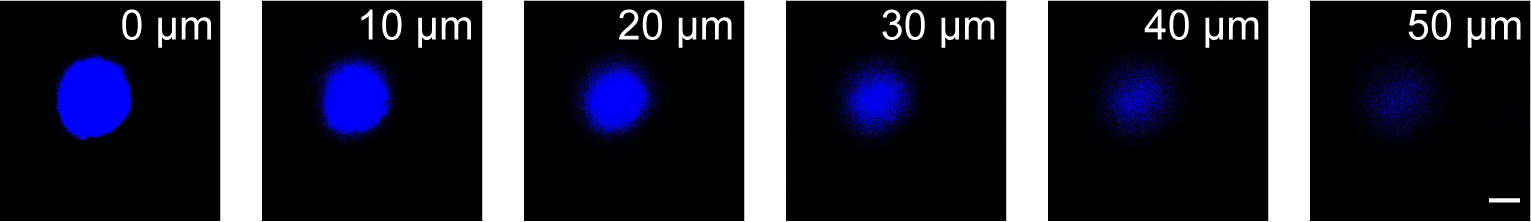


Figure S15. Z-stack images of phantom of PTMM NPs with intervals of 10 μm. Scale bar: 200 μm.


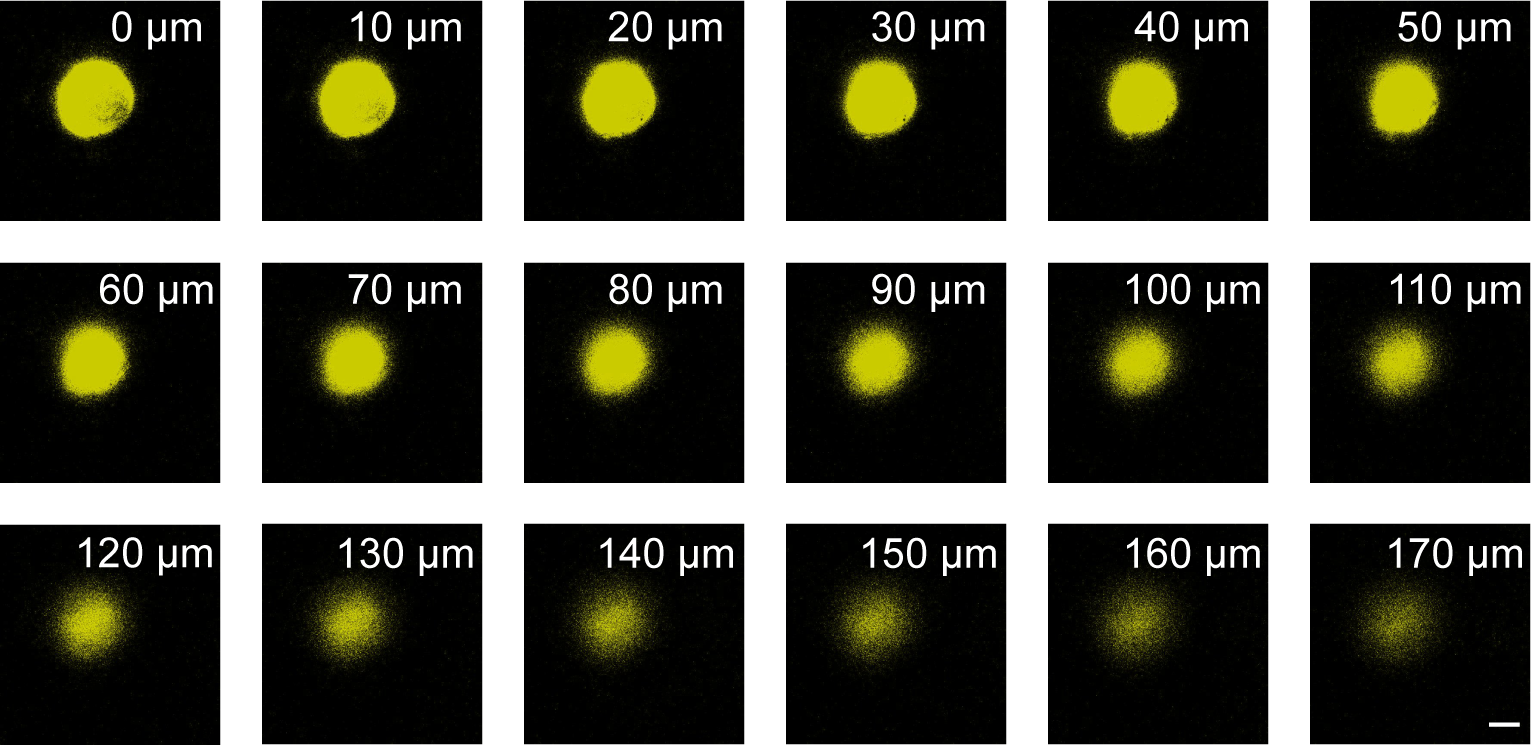


Figure S16. Z-stack images of phantom of TTNA NPs with intervals of 10 μm. Scale bar: 200 μm.


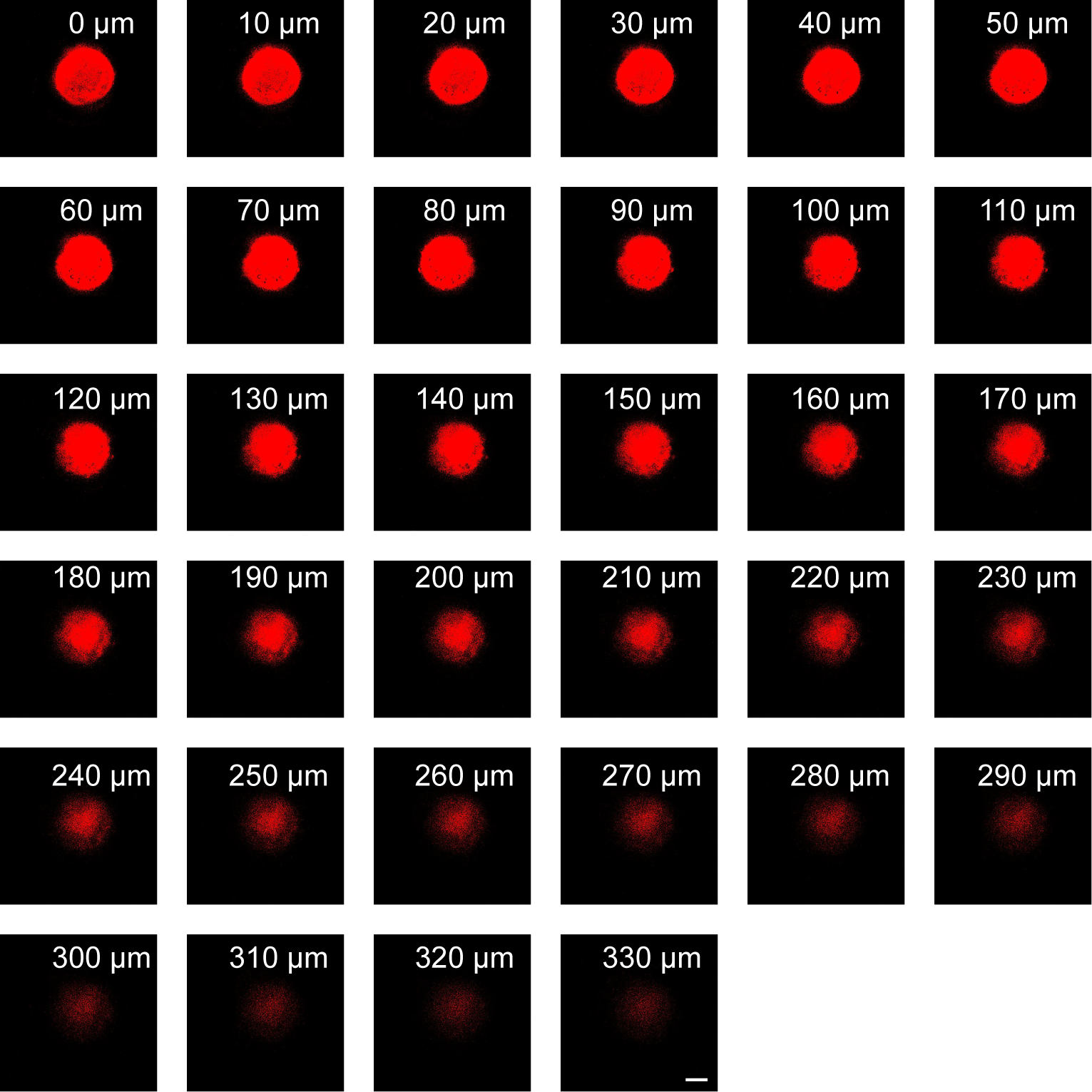


Figure S17. Z-stack images of phantom of TTBI NPs with intervals of 10 μm. Scale bar: 200 μm.


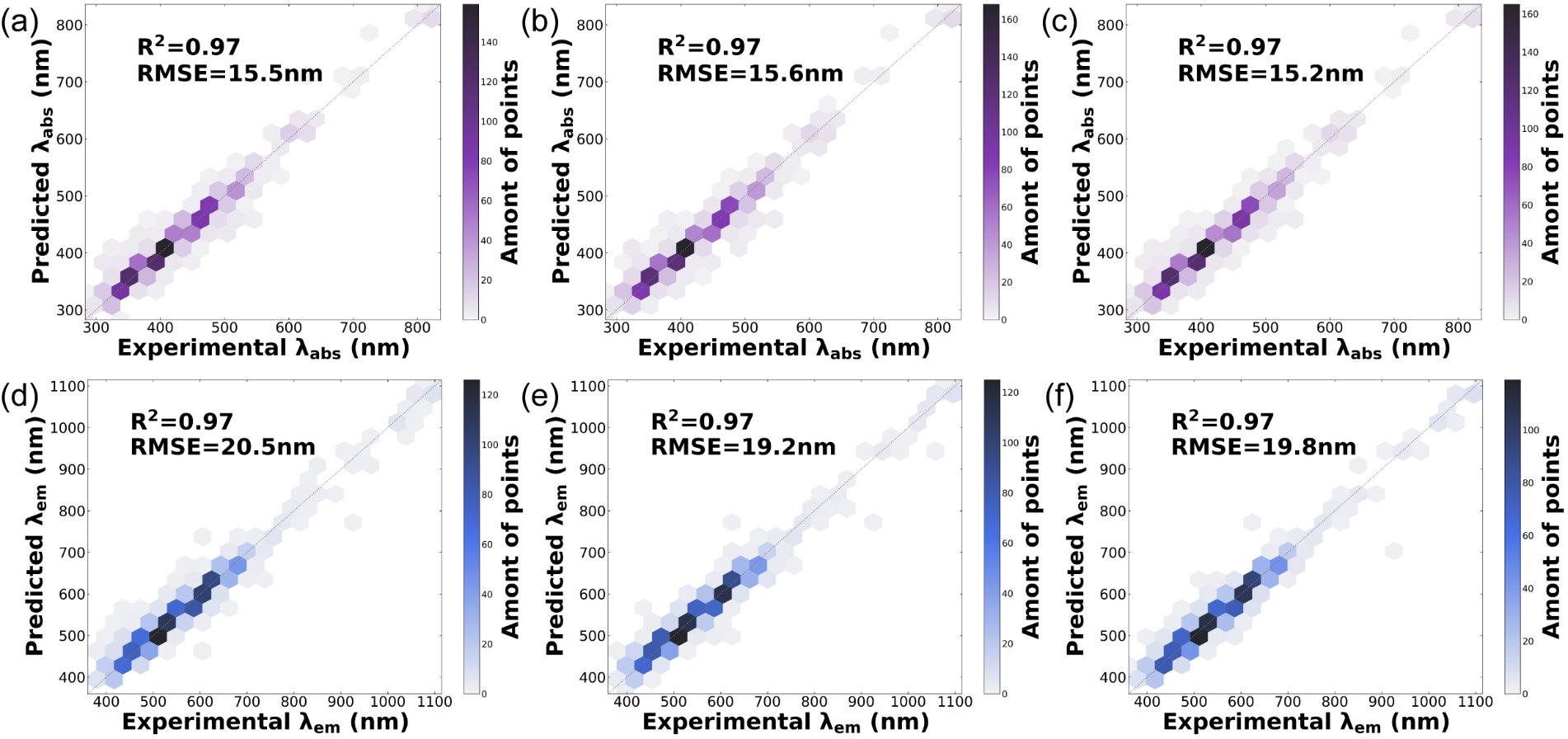


Figure S18. Experimental and predicted data are compared using 10-fold cross-validation. The XGB model predicts λ_abs_ based on various multi-modal descriptors, including (a) morgan-daylight fingerprint, (b) atom-pair-daylight fingerprint, and (c) toptorsion-daylight fingerprint. The CNN model predicts λ_em_ based on various multi-modal descriptors, including (d) morgan-daylight fingerprint, (e) atom-pair-daylight fingerprint, and (f) toptorsion-daylight fingerprint.


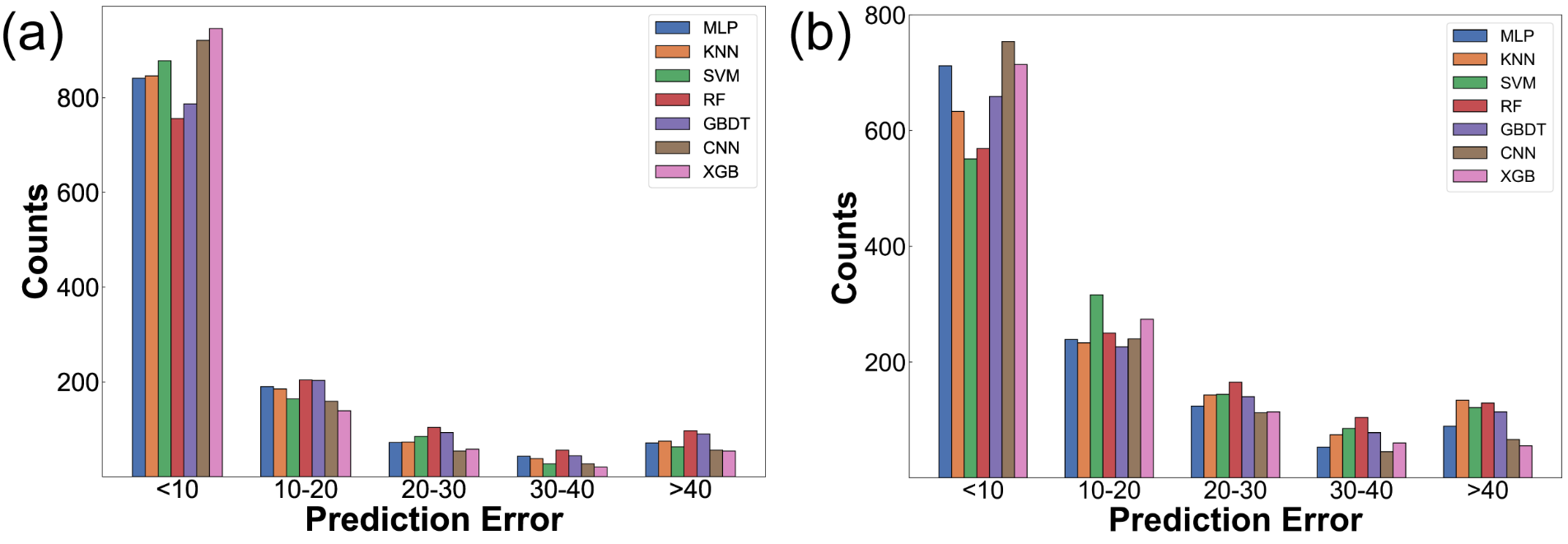


Figure S19. ML prediction error distribution. (a) Absorption peak error distribution. (b) Emission peak error distribution


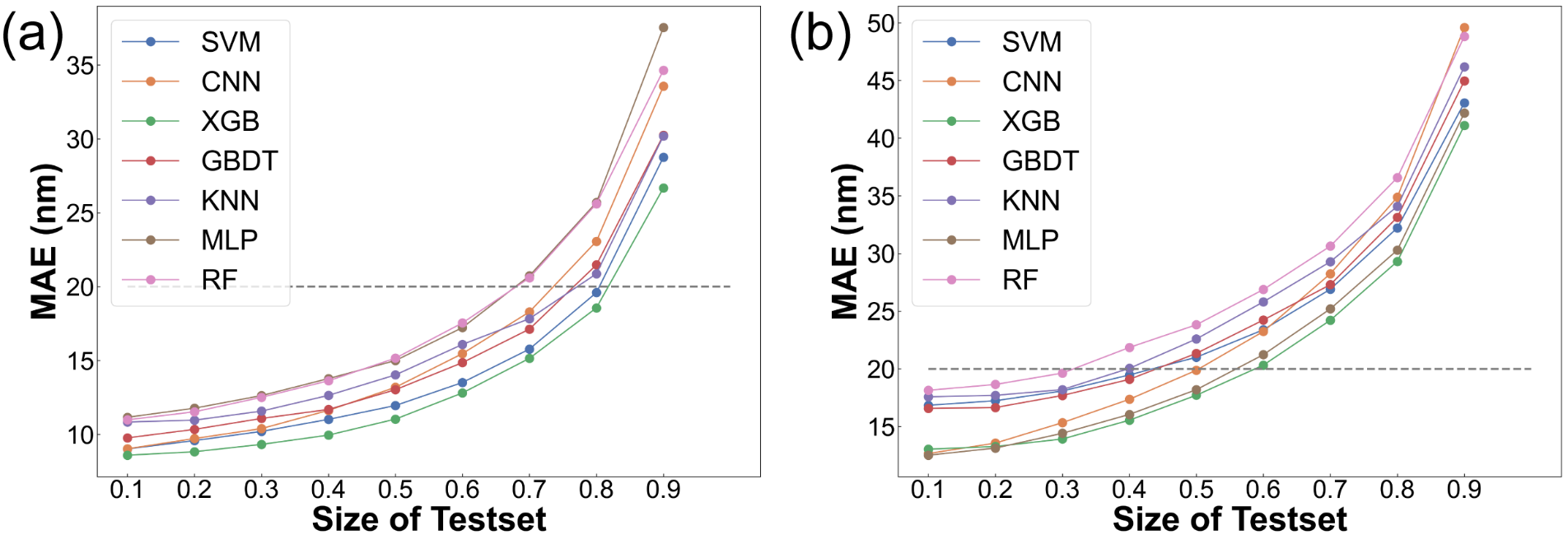


Figure S20. Model scalability. (a) Absorption model scalability. (b) Emission model scalability.


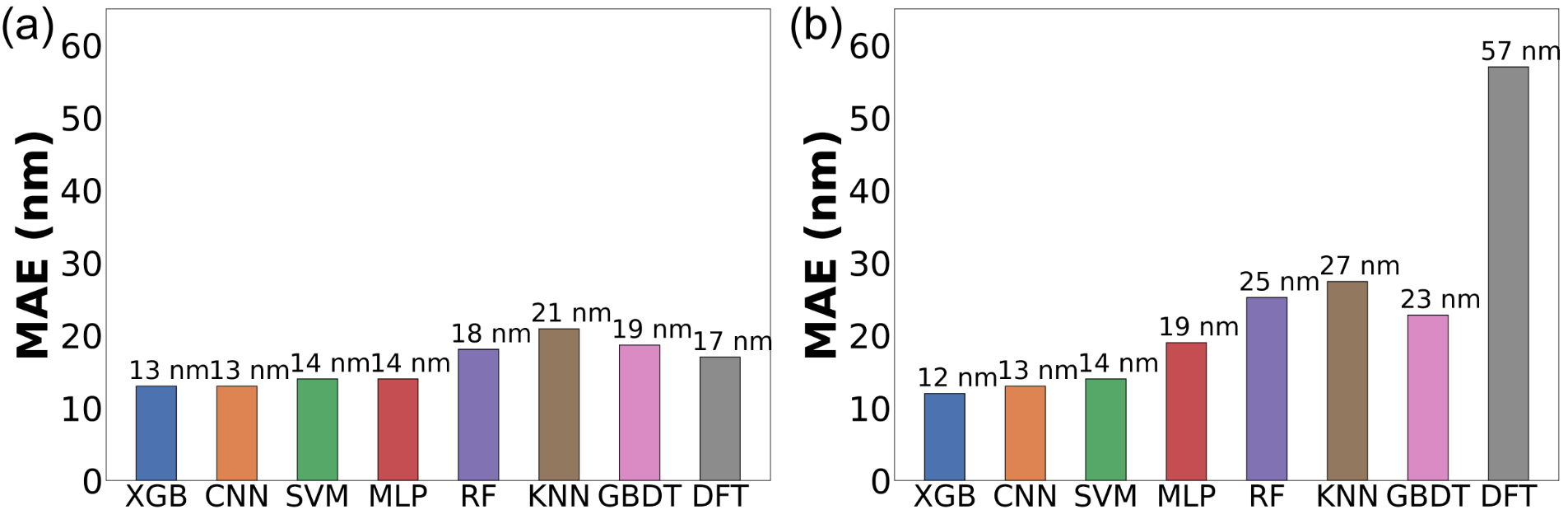


Figure S21. Comparison of ML accuracy and TD-DFT. (a) MAE of predicted absorption peak. (c) MAE of predicted emission peak.


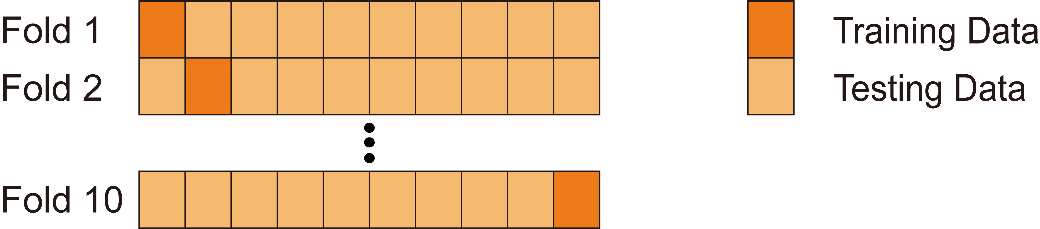


Figure S22. Illustration of 10-fold cross-validation.
